# STAT4 drives optimal expansion and transcriptional repression of type I interferon pathway in inflammatory ILC2

**DOI:** 10.1007/s00018-026-06157-6

**Published:** 2026-03-26

**Authors:** Giuseppe Pietropaolo, Gianluca Scarno, Arianna Maria Candelotti, Giordana Garzillo, Chiara Di Censo, Giovanna Peruzzi, Cinzia Fionda, Helena Stabile, Francesca Sozio, Chiara D’Aquino, Rosa Molfetta, Fabrizio Leone, Bianca Laura Cinicola, Alessandra Gori, Cecilia Ciancaglini, Silvia Santopolo, Linda Quatrini, Anna Maria Zicari, Han-Yu Shih, Yohei Mikami, Angela Gismondi, Giovanni Bernardini, Katharine C. Hsu, Angela Santoni, Silvano Sozzani, Mattia Laffranchi, Giuseppe Sciumè

**Affiliations:** 1 Department of Molecular Medicine, Sapienza University of Rome, 00161 Rome, Italy; 2 Laboratory Affiliated to Istituto Pasteur Italia, Fondazione Cenci Bolognetti, 00161 Rome, Italy; 3 IRCCS Neuromed, 86077 Pozzilli, IS Italy; 4 Human Oncology and Pathogenesis Program, Memorial Sloan Kettering Cancer Center, New York, NY USA; 5 Center for Life Nano- & Neuro-Science, Istituto Italiano Di Tecnologia, Rome, Italy; 6 Department of Maternal Infantile and Urological Sciences, Sapienza University of Rome, 00161 Rome, Italy; 7 Department of Translational and Precision Medicine, Sapienza University of Rome, 00161 Rome, Italy; 8 Innate Lymphoid Cells Unit, Bambino Gesù Children’s Hospital, IRCCS, Rome, Italy; 9 Neuro-Immune Regulome Unit, National Eye Institute, NIH, Bethesda, MD USA; 10 Division of Gastroenterology and Hepatology, Department of Internal Medicine, Keio University School of Medicine, Tokyo, Japan; 11 Immunology Program, Sloan Kettering Institute, Memorial Sloan Kettering Cancer Center, New York, NY USA; 12 Department of Medicine, Weill Cornell Medical College, New York, NY USA

**Keywords:** Innate lymphoid cells, JAK/STAT pathway, Transcriptional regulation, Cytokines, Interferons

## Abstract

Innate immune cells respond rapidly to environmental cues through signal-regulated transcription factors (SRTFs) that sense changes in the tissue microenvironment. Signal transducer and activator of transcription (STAT) proteins are critical regulators of cytokine signaling and determine polarized immune responses. Herein, we reveal that activated type 2 innate lymphocytes (ILC2s) express STAT4, a SRTF canonically linked to type 1 immunity. STAT4 expression is induced in ILC2s upon activation by the alarmin IL-25 and linked with accumulation of lung inflammatory ILC2s (iILC2s). Despite elevated STAT4 expression, iILC2s do not acquire type 1 features, such as interferon (IFN)-γ production or T-bet expression and do not respond to IL-12 stimulation. Instead, STAT4 is activated by type I IFNs and supports the maintenance of the iILC2 pool. Transcriptomic analysis of *Stat4*-deficient ILC2s reveals enhanced type I IFN signaling and impaired proliferation, suggesting that STAT4 functions to antagonize IFN-driven suppression. Our data uncover a novel regulatory axis in which IL-25-induced STAT4 expression equips ILC2s to modulate interferon responses and to prevent aberrant autocrine function of type I IFNs, thus sustaining inflammatory effector populations during immune activation. These findings broaden the understanding of ILC2 activation and suggest new avenues for modulating innate lymphocytes in inflammatory diseases.

**Supplementary Information:**

The online version contains supplementary material available at 10.1007/s00018-026-06157-6.

## Introduction

Signal-regulated transcription factors (SRTFs) enable immune cells to sense environmental cues and trigger context-specific transcriptional programs [1]. Among SRTFs, members of the Janus kinase (JAK) and signal transducer and activator of transcription (STAT) family play pleiotropic roles in key organismal processes and direct several aspects of both adaptive and innate immune responses [2]. Studies on T cell differentiation have established several paradigms for STAT-dependent immune regulation. Distinct STAT family members drive lineage specification in T helper (Th) cells, STAT4 promotes Th1, STAT6 supports Th2, and STAT3 induces Th17 differentiation, while concurrently repressing alternative fates [2–4]. Notably, STAT expression is highly dynamic during an immune response and fine-tuned expression of these TFs allows functional diversity and plasticity of immune cells [5].

In contrast to T cells, the effector differentiation of innate lymphocytes, namely natural killer (NK) cells and innate lymphoid cells (ILCs), is mainly driven by lineage defining transcription factors (LDTFs), while STAT proteins, which are often expressed at high levels even at steady state, are key players of their activation [6–8]. Indeed, NK cells and type 1 ILCs (ILC1) can still develop in the absence of STAT4, while type 2 ILCs (ILC2s) do not require STAT6 for development [9–11]. Similar to T cells, STAT expression in innate lymphocytes can be dynamically regulated during immune activation. For instance, NK cells upregulate STAT1 and STAT5 during viral infection, supporting type I interferon (IFN) responses and optimal viral control [12, 13].

At the transcriptional and functional level, ILC2s are defined by expression of the LDTF GATA-3, and are activated by the epithelial-derived cytokines/alarmins IL-25 and IL-33, which drive type 2 cytokine production [14–16]. Administration of IL-25 or infection with *Nippostrongylus brasiliensis* in mice induces migration of intestinal ILC2s to the lung, where these cells give rise to a distinct population termed inflammatory ILC2s (iILC2s) [17–19]. These cells differ from lung-resident natural ILC2s (nILC2s) by their ability to produce the type 3 cytokine IL-17 [18].

ILC2 function is tightly regulated by STAT signaling pathways. While STAT6 is dispensable for ILC2 development, it is essential for their activation and protection against *Alternaria alternata* infection [10, 11]. Additionally, STAT3 is upregulated during ILC2 activation downstream of IL-33 signaling and contributes to regulating their effector functions in the context of ILC2-driven allergic inflammation [20]. In contrast, STAT1, acting downstream of interferons and IL-27, plays a pivotal negative regulatory role by limiting ILC2 proliferation and cytokine production upon activation [21–23]. These findings underscore the importance of dissecting cytokine/STAT signaling axes in the regulation of ILC2 responses.

Herein, we found that ILC2s upregulate STAT4 upon acute activation downstream of IL-25, both in vitro and in vivo. Specifically, STAT4 expression is hardwired in the iILC2 epigenetic and transcriptional program, yet its function is uncoupled from T-bet expression and conventional IFN-γ-response. Moreover, we found that STAT4 activation occurs independently of IL-12, while IFN-β induces robust STAT4 phosphorylation. Through genetic and transcriptomic analyses, we showed that STAT4 is required for optimal expansion of iILC2s in a cell intrinsic manner. Mechanistically, loss of *Stat4* leads to impaired ILC2 proliferation, heightened type I IFN signaling and autocrine function of type I IFNs, highlighting a key role for STAT4 in coordinating the balance between iILC2 expansion and type I IFN responses.

## Results

### ILC2s are poised for STAT4 expression

**Fig. 1:**
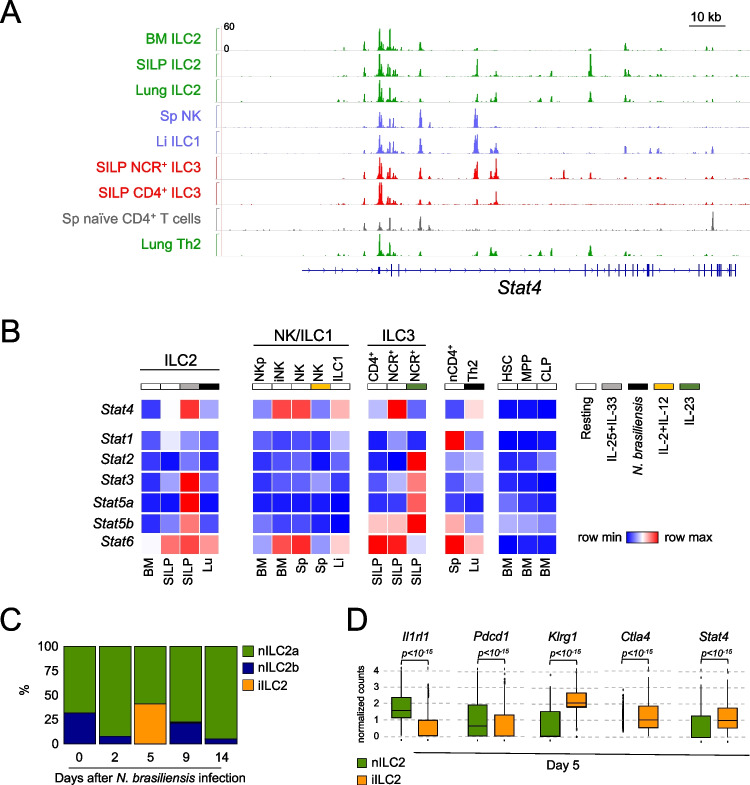
Acute activation increases *Stat4* transcript levels in ILC2s. (**A**) Genomic snapshot shows normalized ATAC-seq signals at the *Stat4* locus in ILC2s isolated from the bone marrow (BM) and small intestinal lamina propria (SILP) of untreated mice; ILC2s isolated from the lung of *N. brasiliensis* infected mice; splenic (Sp) NK cells; liver (Li) ILC1s; SILP NCR^+^ and CD4^+^ ILC3s; Sp naïve CD4^+^ T cells; Th2 isolated from the lung of *N. brasiliensis* infected mice. (**B**) Heatmap shows expression of genes encoding for STATs in ILC2s, NK cells, including NK cell precursors (NKp), and immature NK cells (iNK), ILC1s, and ILC3s from distinct tissues and activation states. Hematopoietic stem cells (HSC), multipotent progenitors (MPP), common lymphoid precursors (CLP) are also added as negative controls. (**C**) Frequency of nILC2 and iILC2 clusters from the lung of *N. brasiliensis* infected mice (day 2, 5, 9, and 14 post-infection) or untreated mice (day 0) was analyzed by scRNA-seq. Identity of nILC2 and iILC2 clusters was defined based on the differential expression of known markers. (**D**) Box plots show the expression of *Il1rl1*, *Pdcd1*, *Klrg1*, *Ctla4* and *Stat4* in nILC2s and iILC2s at day 5 post-infection (see Suppl. Figure 1 and Suppl. Table 1 for additional details). The *p* value was calculated using the Wilcoxon test

Under steady-state conditions, murine type 1 innate lymphocytes and NCR⁺ ILC3s exhibit high levels of STAT4 expression, which is critical for promoting type 1 immune responses [9, 12, 24]. To determine whether ILC2s can also express STAT4, we first mined ATAC-seq and RNA-seq data of cells isolated from various tissues and activation states [25]. In resting ILC2s isolated from the small intestine lamina propria (SILP), we observed several accessible regulatory elements within the *Stat4* locus, indicating potential for gene expression (Fig. 1A). A similar DNA accessibility profile was detected in lung ILC2s from *Nippostrongylus brasiliensis*-infected mice (at day 10 post-infection), whereas bone marrow (BM) ILC2s exhibited limited accessibility at the *Stat4* locus (Fig. 1A). These profiles suggest tissue-specific or developmental regulation of *Stat4* chromatin accessibility in ILC2s. Moreover, most of the accessible regions in ILC2s overlapped with those found in ILC1 and NCR^+^ ILC3, which both express this TF at high levels, as well as with regulatory regions accessible in Th2 cells (Fig. 1A). These results support the hypothesis that ILC2s possess the potential to express *Stat4*, similar to other type 1 innate lymphocytes.

Consistent with the DNA-accessibility profile, we detected *Stat4* transcripts in resting SILP ILC2s and observed that expression increased to levels comparable to those found in type 1 innate lymphocytes and NCR^+^ ILC3 upon activation with the alarmins IL-25 and IL-33 (Fig. 1B). *Stat4* was also expressed by Th2 cells, suggesting potential shared regulatory mechanisms with ILC2s. Following cytokine stimulation, intestinal ILC2s also expressed high levels of *Stat3* and *Stat5a/b* (Fig. 1B), which have been previously linked to survival and differentiation of these cells [20, 26]. These findings suggest that acute activation enables ILC2s to modify their cytokine responsiveness through regulation of STAT expression, such as *Stat4*.

To further validate these observations in vivo, we analyzed scRNA-seq data of lung adaptive and innate immune cells isolated from mice infected with *N. brasiliensis* [27], a model known to elicit generation of iILC2s in the lung [17]. ILC2s were identified by the expression of lineage-defining markers and subsequently reclustered, distinguishing two clusters of nILC2s (nILC2a and nILC2b) as well as a distinct iILC2 cluster (Suppl. Figure 1A). The latter peaked at day 5 post-infection before rapidly declining (Fig. 1C). The identity of iILC2s was confirmed by reduced expression of *Il1rl1* (encoding the IL-33 receptor), *Pdcd1* (PD-1), *Il7r* and *Il5*, along with upregulation of *Klrg1*, *Il17a* and *Ctla4* (Fig. 1D and Suppl. Figure 1B). Consistent with transcriptomic data from cytokine-stimulated cells, *Stat4* transcripts were also detected in vivo in ILC2s, with significantly higher expression in iILC2s compared to nILC2s (Fig. 1D).

Collectively, these findings demonstrate that ILC2s are epigenetically and transcriptionally poised for STAT4 expression and that this TF can be induced upon activation, both in vitro and in vivo.

### IL-25 is a major inducer of STAT4 in ILC2s

**Fig. 2:**
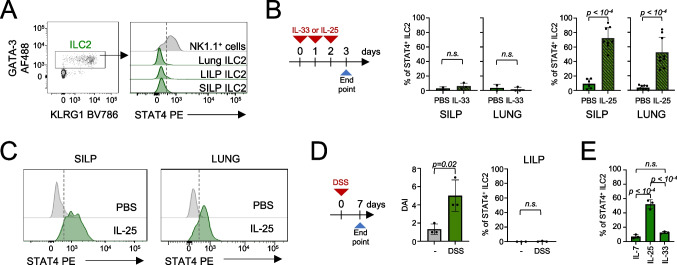
STAT4 expression in ILC2s in distinct inflammation settings. (**A**) Flow cytometry histogram plot shows the expression levels of total STAT4 in ILC2s (gated as lin^−^GATA-3^+^ cells) from SILP, LILP and lung. NK1.1^+^ cells, including NK cells and ILC1s, from the intestine were used as control. A representative experiment (*n* = 3) of two independent experiments performed is shown. Full gating strategy in Suppl. Figure 2A. (**B**) Mice were administered with IL-33 (200 ng/mouse) or IL-25 (200 ng/mouse) for three consecutive days or PBS, as control. Histogram plots depict the percentage of total STAT4^+^ ILC2s. A representative experiment (*n* = 3) of two independent experiments performed is shown, for experiments with IL-33 administration. Pooled data from at least three independent experiments are shown for experiments with IL-25 administration. Each dot represents an individual mouse, and the bar depicts the mean with s.e.m. (**C**) Representative flow cytometry histogram plots depict STAT4 expression in ILC2s isolated from SILP or lung of control and IL-25-treated mice. (**D**) Cells were isolated from the LILP of DSS-treated (day 7) mice. Plots show Disease Activity Index (DAI) and the frequency of STAT4^+^ cells among LILP ILC2s from control and DSS-treated mice. A representative experiment of two independent experiments performed is shown (*n* = 3). Each dot represents an individual mouse and the bar depicts the mean with s.e.m. (**E**) Histogram plot shows the frequency of STAT4^+^ ILC2s after ex vivo stimulation of SILP cells with IL-25, IL-33 or IL-7 alone, for 15 h. A representative experiment (*n* = 3) of two independent experiments performed is shown. Each dot represents an individual mouse and the bar depicts the mean with s.e.m. For statistical analysis, unpaired Student’s t test was used in panel B and D, one-way ANOVA with post-hoc Tukey HSD for panel E

Based on the results obtained by DNA-accessibility and transcriptomic analysis, we next investigated whether STAT4 can be expressed at the protein level in ILC2s. Despite the accessibility of *Stat4* locus, resting SILP ILC2s expressed minimal levels of STAT4 protein as compared to type 1 cells (Fig. 2A; gating strategy in Suppl. Figure 2A). Similar low expression levels were observed in ILC2s isolated from the large intestinal lamina propria (LILP) and the lung (Fig. 2A). To determine whether inflammatory cues can induce STAT4 expression in vivo, mice were treated with the alarmins IL-33 or IL-25 via intraperitoneal injection for three consecutive days. These experimental conditions are commonly employed to activate ILC2s in the intestine and lung [17]. Following IL-33 treatment, STAT4 expression remained unchanged in ILC2s isolated from either the SILP or the lung (Fig. 2B). We obtained similar results from ILC2s isolated from the mesenteric lymph nodes and large intestine (Suppl. Figure 2B). In contrast, SILP and lung ILC2s from IL-25-treated mice significantly increased STAT4 levels compared to ILC2s from untreated controls (Fig. 2B and C). These results suggest that STAT4 induction in ILC2s is selectively triggered by IL-25 and is not a general consequence of ILC2 activation. To further test this hypothesis, we next challenged mice with dextran sodium sulfate (DSS) in the drinking water for seven days to induce inflammation in the LILP. Unlike the response observed following IL-25 administration, ILC2s isolated from the LILP of DSS-treated mice did not upregulate STAT4 (Fig. 2D). On the other hand, ex vivo stimulation of SILP ILC2s with IL-25 resulted in a marked upregulation of STAT4 expression compared with cells cultured with IL-7 or IL-33 alone (Fig. 2E), corroborating the predominant role of IL-25 in STAT4 induction.

Taken together, these data highlight a context-dependent regulation of the expression of STAT4 in ILC2s, which can be acquired upon activation in response to IL-25.

### STAT4 is induced in iILC2s in the absence of type 1 functional conversion

**Fig. 3:**
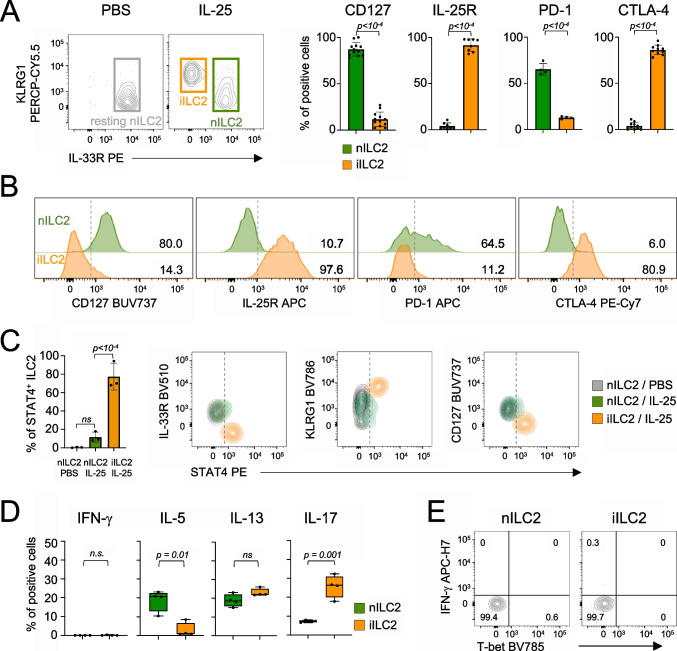
STAT4 is upregulated in iILC2s from the lung. (**A**) Mice were administered with IL-25 or PBS, as control. Cells were isolated from the lung; nILC2s and iILC2s were defined as IL33R^+^ and KLRG1^+^IL-33R^−^ cells, respectively. Cell identity was validated by the expression of CD127, IL-25R, PD-1 and CTLA-4. Pooled data from at least three independent experiments are shown. Each dot represents an individual mouse, and the bar depicts the mean with s.e.m. (**B**) Representative flow cytometry histogram plots for CD127, IL-25R, PD-1 and CTLA-4 expression in lung nILC2s and iILC2s of IL-25 treated mice. Dotted lines represent the background signal. (**C**) STAT4 expression was evaluated in lung nILC2s and iILC2s of IL-25 treated mice and from resting nILC2s from mice administered with PBS, by flow cytometry. A representative experiment (*n* = 3) of three independent experiments performed is shown. Each dot represents an individual mouse, and the bar depicts the mean with s.e.m. (**D**) Box plots show percentage of positive cells for IFN-γ, IL-5, IL-13 and IL-17 in lung ILC2s from IL-25 treated mice upon ex vivo restimulation with PMA/Ionomycin for three hours. A representative experiment (*n* = 4) of two independent experiments performed is shown. Each dot represents an individual mouse. Median and quartiles are shown. (**E**) Expression of T-bet and IFN-γ in lung nILC2s and iILC2s from IL-25 treated mice. A representative experiment (*n* = 3) of two independent experiments performed is shown. For statistical analysis, unpaired Student’s t test (panel A and D) and one-way ANOVA with post-hoc Tukey HSD (panel C) were used

Given that IL-25 administration promotes differentiation and migration of iILC2s in the lung similarly to *N. brasiliensis* infection, whereas IL-33 and DSS fail to expand this population [18, 28], we hypothesized that the differential STAT4 expression observed across these models may be linked to iILC2 development. To test this hypothesis, we administered IL-25 to mice and identified lung iILC2s and nILC2s based on the differential expression of KLRG1, IL-33R or CD127 (Fig. 3A and Suppl. Figure 2A). Similarly to the iILC2s generated upon *N. brasiliensis* infection, IL-25-induced iILC2s showed suppression of CD127 and PD-1 expression as well as induction of IL-25R and CTLA-4 (Figs. 3A and B). Under these conditions, the majority of lung iILC2s expressed higher levels of STAT4 compared to both nILC2s from IL-25-treated and naive ILC2s from control mice (Fig. 3C). These findings indicate that upregulation of STAT4 expression in ILC2s is associated with generation of the iILC2 subset.

Plasticity of ILC2s towards the ILC1 fate can occur through a process driven by IL-12 and IL-1β [29–31]. We therefore investigated whether the expression of STAT4 in iILC2s could confer these cells the ability to acquire type 1 features following IL-25 stimulation in vivo. In this model, iILC2s are characterized by a mixed type 2/type 3 cytokine profile defined by the expression of IL-13 and IL-17 (Fig. 3D and Suppl. Figure 2C). Despite STAT4 expression, iILC2s isolated from IL-25-treated mice did not gain the ability to produce IFN-γ (Figs. 3D and E). Furthermore, these cells failed to express T-bet (Fig. 3E), indicating that STAT4 expression in iILC2s is not sufficient to initiate a canonical type 1 transcriptional program.

Taken together, our results indicate that STAT4 is hardwired in the differentiation program of iILC2s and that its function is uncoupled from T-bet expression and the canonical acquisition of type 1 effector functions, such as IFN-γ production.

### *Stat4* deletion impairs the pool of iILC2s

**Fig. 4:**
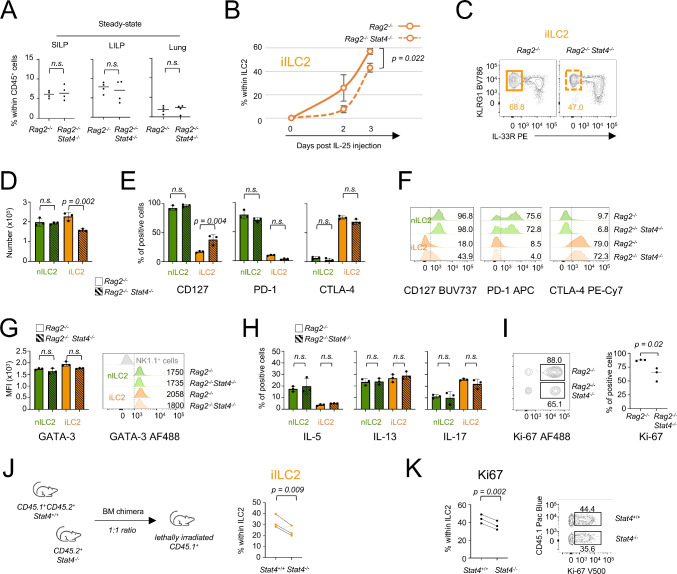
STAT4 controls optimal iILC2 expansion. (**A**) Frequency of ILC2s in the SILP, LILP and lung from untreated *Rag2*^*−/−*^ and *Rag2*^*−/−*^*Stat4*^*−/−*^ mice. A representative experiment (*n* = 4) of two independent experiments performed is shown. Each dot represents an individual mouse and the bar depicts the median. (**B**) *Rag2*^*−/−*^ and *Rag2*^*−/−*^*Stat4*^*−/−*^ mice were administered with IL-25 (200 ng/mouse) for 2 and 3 days. Cells were isolated from the lung at different time points and frequency of iILC2s was established by flow cytometry. The plot depicts the frequency of iILC2s in *Rag2*^*−/−*^ and *Rag2*^*−/−*^*Stat4*^*−/−*^ mice. Bars depict the mean with s.e.m. (**C**) Representative flow panel of iILC2s from *Rag2*^*−/−*^ and *Rag2*^*−/−*^*Stat4*^*−/−*^ mice, at 3 days after IL-25 administration. (**D**) The plot depicts the numbers of nILC2s and iILC2s in *Rag2*^*−/−*^ and *Rag2*^*−/−*^*Stat4*^*−/−*^ mice, at 3 days after IL-25 administration. A representative experiment of two independent experiments is shown. Each dot represents an individual mouse and the bar depicts the mean with s.e.m. (**E**) Histogram plots depict the frequency of CD127^+^, PD-1^+^ and CTLA-4^+^ cells in nILC2 and iILC2 from *Rag2*^*−/−*^ and *Rag2*^*−/−*^*Stat4*^*−/−*^ mice, at 3 days after IL-25 administration. A representative experiment of two independent experiments is shown. Each dot represents an individual mouse and the bar depicts the mean with s.e.m. (**F**) Representative flow cytometry histogram plots for CD127, PD-1 and CTLA-4 expression in lung nILC2s and iILC2s from IL-25 treated *Rag2*^*−/−*^ and *Rag2*^*−/−*^*Stat4*^*−/−*^ mice. (**G**) Left panel, GATA-3 expression in lung nILC2s and iILC2s from IL-25 treated *Rag2*^*−/−*^ and *Rag2*^*−/−*^*Stat4*^*−/−*^ mice, evaluated by flow cytometry. Each dot represents an individual mouse and the bar depicts the mean with s.e.m. Right panel, representative flow cytometry histogram plot for GATA-3. (**H**) Plots show percentage of positive cells for IL-5, IL-13 and IL-17 in lung ILC2s from IL-25 treated *Rag2*^*−/−*^ and *Rag2*^*−/−*^*Stat4*^*−/−*^ mice upon ex vivo restimulation with PMA/Ionomycin for three hours. A representative experiment (*n* = 3) of two independent experiments performed is shown. Each dot represents an individual mouse and the bar depicts the mean with s.e.m. (**I**) Concatenate flow cytometry plot and scatter plot depict the percentage of Ki-67^+^ cells among ILC2s generated from *Stat4*^+*/*+^ and *Stat4*^*−/−*^ mice after IL-25 administration. Each dot represents an individual mouse and the bar depicts the median. A representative experiment of two performed is shown. (**J**) Bone marrow cells from *Stat4*^+*/*+^ (CD45.1^+^/CD45.2^+^) and *Stat4*^*−/−*^ (CD45.2^+^) mice were transferred into lethally irradiated recipients (CD45.1^+^) mice. After reconstitution, ILC2s were analyzed 2 days after IL-25 administration. The plot depicts the frequency of iILC2s among ILC2s generated from *Stat4*^+*/*+^ and *Stat4*^*−/−*^ mice. A representative experiment of two performed is shown. (**K**) Plots depict the percentage of Ki-67^+^ cells among ILC2s generated from *Stat4*^+*/*+^ and *Stat4*^*−/−*^ mice after IL-25 administration. A representative experiment of two performed is shown. For statistical analysis, unpaired Student’s t test (panel A, B, and I), paired Student’s t test (panel J and K), and one-way ANOVA with post-hoc Tukey HSD (panel D, E, G and H) were used

Since STAT4 antagonizes Th2 differentiation [32], we hypothesized that expression of this TF in ILC2s might serve as a regulatory checkpoint upon activation. To test this hypothesis in a system devoid of confounding effects of STAT4 on T cell responses, we analyzed ILC2s in *Rag2*^*−/−*^ and *Rag2*^*−/−*^*Stat4*^*−/−*^ mice. Consistent with the minimal STAT4 expression in resting ILC2s, germline deletion of *Stat4* did not affect the homeostatic pool of ILC2s in intestine and lung (Fig. 4A). Next, we challenged both *Rag2*^*−/−*^ and *Rag2*^*−/−*^*Stat4*^*−/−*^ mice with IL-25 and tracked the presence of iILC2s in the lung after treatment. Under these conditions, the frequency (Figs. 4B and C) and number (Fig. 4D) of iILC2s were impaired in *Rag2*^*−/−*^*Stat4*^*−/−*^ mice, suggesting that, contrary to its established antagonistic role in Th2 cells, STAT4 can support the iILC2 pool following IL-25 stimulation.

To further dissect the role of STAT4 in regulating iILC2 phenotype and function, we first assessed the expression of markers discriminating nILC2s and iILC2s (as established in Fig. 3A). We found that iILC2s from *Rag2*^*−/−*^*Stat4*^*−/−*^ mice failed to efficiently downregulate CD127 expression compared with *Stat4*-sufficient ILC2s (Fig. 4E and F). In contrast, neither the frequencies of CTLA-4- and PD-1-expressing cells (Fig. 4E and F) nor GATA-3 expression levels (Fig. 4G) were significantly different between the two groups, indicating that *Stat4* deletion delays CD127 downregulation without impairing overall ILC2 identity. At a functional level, the absence of *Stat4* did not affect the ability of ILC2s to produce cytokines, since iILC2s maintained similar potential to produce IL-13 and IL-17 (Fig. 4H). By contrast the percentage of Ki-67^+^ cells was reduced in *Stat4*-deficient ILC2s (Fig. 4I), suggesting that STAT4 contributes to the maintenance of ILC2 numbers by sustaining their proliferative capacity.

To determine whether the effect of STAT4 is cell-intrinsic, we generated mixed bone marrow chimeras by transferring equal ratios of bone marrow cells from *Stat4*^+*/*+^ (CD45.1^+^/CD45.2^+^) and *Stat4*^*−/−*^ (CD45.2^+^) mice into lethally irradiated recipients (CD45.1^+^). Following IL-25 stimulation in vivo, the ratio of CD45.2^+^ (*Stat4*^+*/*+^) and CD45.1^+^CD45.2^+^ (*Stat4*^*−/−*^) cells was comparable (Suppl. Figure 2D); while, consistent with the results we obtained in *Rag2*^*−/−*^*Stat4*^*−/−*^ mice, *Stat4*-deficient bone marrow cells gave rise to fewer iILC2s (Fig. 4J). This reduced frequency was accompanied by a lower proportion of Ki-67-expressing cells in *Stat4*-deficient ILC2s compared with wild type cells, implying a proliferative disadvantage (Fig. 4K).

Together, these results indicate that STAT4 sustains the IL-25-driven expansion of iILC2s in the lung and is required for optimal proliferation in a cell intrinsic manner.

### STAT4 is activated by type I IFNs, but not IL-12, and limits type I IFN transcriptome in iILC2s

**Fig. 5:**
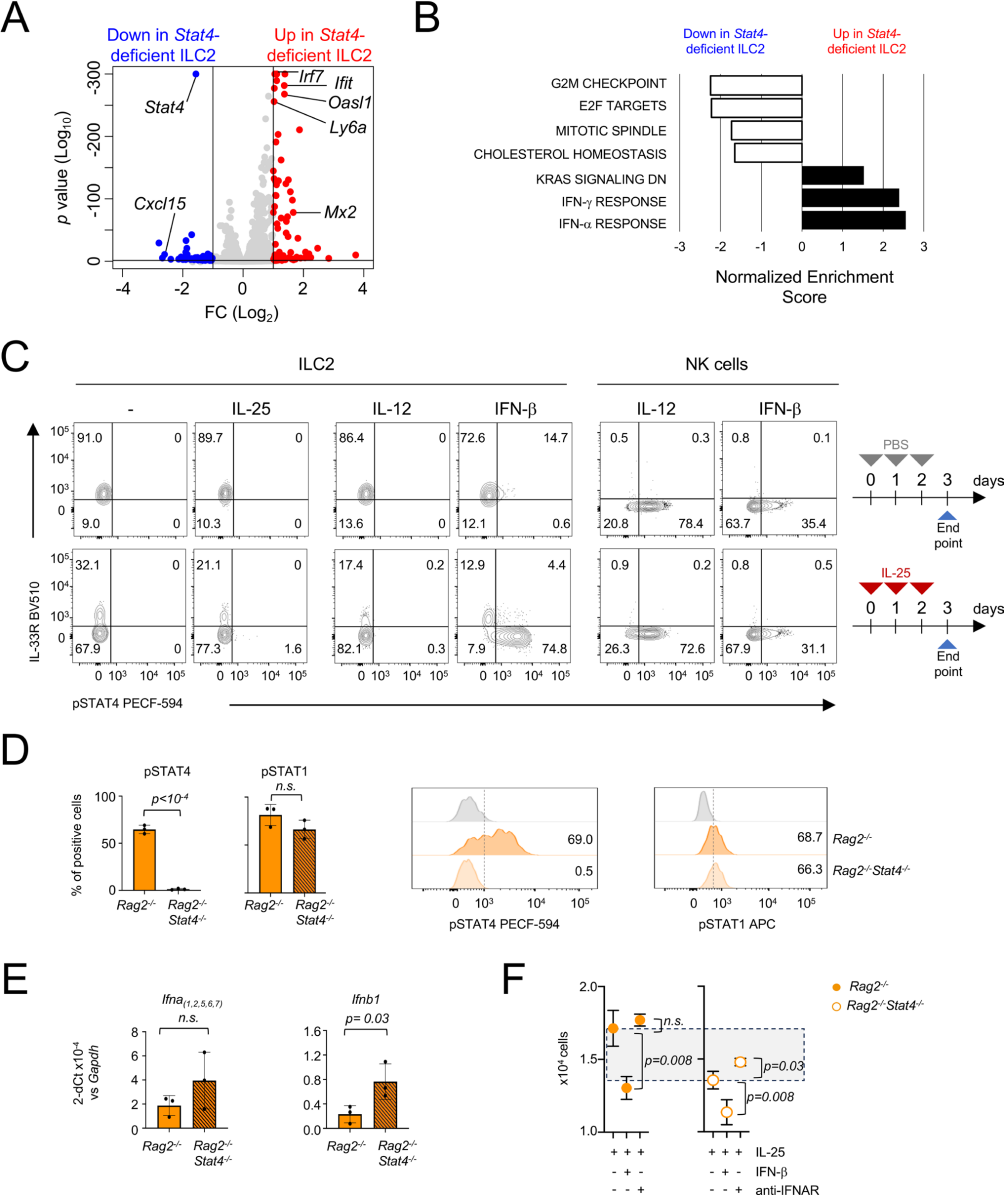
STAT4 constrains type I IFN transcriptome in ILC2s. (**A**) *Rag2*^*−/−*^ and *Rag2*^*−/−*^*Stat4*^*−/−*^ mice were administered with IL-25 for 3 days. iILC2s were sorted as KLRG1^hi^ cells (Suppl. Figure 3A) and processed for bulk-RNA-seq. Volcano plot highlights upregulated and downregulated genes (|Log_2_FC|> 1; adjusted *p* value < 0.05) in ILC2s from *Rag2*^*−/−*^*Stat4*^*−/−*^ mice compared with cells from *Rag2*^*−/−*^ mice. (**B**) Top hits by GSEA using all MSigDB hallmark gene sets. (**C**) Flow cytometry plots show STAT4 phosphorylation in lung ILC2s from IL-25-treated or control (PBS) mice following ex vivo stimulation with IL-25, IL-12 and IFN-β, for 30 min. NK cells were shown as control. A representative experiment (*n* = 3) of two independent experiments performed is shown. (**D**) Plots showing STAT4 and STAT1 phosphorylation in lung iILC2s from IL-25-treated *Rag2*^*−/−*^ and *Rag2*^*−/−*^*Stat4*^*−/−*^ mice following ex vivo stimulation with IFN-β (10,000 IU/ml) for 30 min. Each dot represents an individual mouse and the bar depicts the mean with s.e.m. A representative experiment (*n* = 3) of two independent experiments performed is shown. (**E**) qRT-PCR analysis of *Ifna* and *Ifnb1* transcripts in FACS-sorted KLRG1^hi^ ILC2 isolated from IL-25-treated *Rag2*^*−/−*^ and *Rag2*^*−/−*^*Stat4*^*−/−*^ mice. Each dot represents an individual mouse and the bar depicts the mean with s.e.m. A representative experiment (*n* = 3) of two independent experiments performed is shown. (**F**) Quantification of ILC2 isolated from IL-25-treated *Rag2*^*−/−*^ and *Rag2*^*−/−*^*Stat4*^*−/−*^ mice and cultured (2 × 10^4^ cells per well) with IL-25, IFN-β (500 IU/ml) and anti-IFNAR antibody (10μg/ml). A representative experiment (*n* = 3) of two independent experiments performed is shown. Grey area highlights the difference between ILC2 numbers from IL-25-treated *Rag2*^*−/−*^ and *Rag2*^*−/−*^*Stat4*^*−/−*^ mice. For statistical analysis, unpaired Student’s t test (panel D, E and F) was used

To elucidate the role of *Stat4* deletion in iILC2s, we analyzed the transcriptome of FACS-sorted KLRG1^hi^ ILC2s isolated from IL-25 treated *Rag2*^*−/−*^ and *Rag2*^*−/−*^*Stat4*^*−/−*^ mice, by bulk RNA-seq (sorting strategy in Suppl. Figure 3A; full list of differentially expressed genes, DEGs, *p* < 0.05; |Log2FC|> 1 in Suppl. Table 1). Differential expression analysis revealed that ILC2s from *Rag2*^*−/−*^*Stat4*^*−/−*^ mice showed a higher number of upregulated genes (91) than downregulated genes (59) relative to *Stat4*-sufficient cells (Fig. 5A). These findings are in agreement with STAT4 functioning mainly as a transcriptional suppressor in ILC2s. However, we noticed that most upregulated genes in *Stat4*-deficient ILC2s were interferon-stimulated genes (ISGs), such as *Oasl1*, *Ifit*, and *Mx2*. Gene Set Enrichment Analysis (GSEA) performed by using all murine MSigDB hallmark gene sets confirmed that the pathways most suppressed by STAT4 were related to IFN-signaling, with the type I and type II IFN-signaling pathways ranking the highest (Fig. 5B). On the other hand, proliferation-associated terms were significantly affected among the downregulated pathways in *Stat4*-deficient iILC2s (Fig. 5B), in agreement with the decreased iILC2 numbers and Ki-67 expression observed in *Stat4*-deficient mice.

Because STAT4 phosphorylation is potently induced by IL-12, type 1 IFNs and IL-23 [33], we next analyzed the expression of the receptor chains for these cytokines in *Stat4*-deficient and sufficient ILC2s, from the bulk RNA-seq dataset. While *Ifnar1* and *Ifnar2* were both expressed and not altered by the loss of *Stat4*, *Il12rb2* and *Il23r* were undetectable in ILC2s of either genotype (Suppl. Figure 3B). These findings suggest that acute activation enables iILC2s to signal through STAT4 downstream of type I IFNs, rather than IL-12. To confirm this hypothesis, we next investigated the ability of IL-25, IL-12 and IFN-β to effectively induce STAT4 phosphorylation. Thirty-minute stimulation with IL-25 did not induce STAT4 phosphorylation in lung ILC2s, regardless of whether cells were isolated from IL-25-treated or untreated mice (Fig. 5C). These results indicate that IL-25 cannot directly activate STAT4 protein in ILC2s.

We next stimulated ILC2s obtained by IL-25 treated mice with IL-12 or IFN-γ. Consistent with the lack of *Il12rb2* expression, IL-12 failed to activate STAT4 in iILC2s (Fig. 5C). As control, IL-12 induced pSTAT4 in lung NK cells from both IL-25-treated or untreated mice (Fig. 5C). The lack of IL-12-dependent STAT4 activation in iILC2s may explain why these cells fail to induce T-bet and IFN-γ, in this model. In contrast to IL-12, IFN-β stimulation resulted in robust STAT4 phosphorylation in iILC2 generated in mice treated with IL-25 (Fig. 5C). This effect was not influenced by culture conditions, as this short-term treatment did not affect total STAT4 protein in iILC2s (Suppl. Figure 3C). Moreover, deletion of *Stat4* did not impact the ability of iILC2s to activate STAT1 upon stimulation with IFN-β, suggesting that type I IFN-responses in these cells can be still elicited by STAT1 (Fig. 5D).

The inhibitory role of STAT4 on the type I IFN pathway has recently been elucidated in NK cells through integrative genome-wide analyses of STAT4 and STAT1 binding and transcriptomic profiling of *Stat4*- and *Stat1*-deficient NK cells during the acute phase of viral infection [34]. This approach identified a discrete cluster of genes directly activated by STAT1 and repressed by STAT4 [34]. Consistent with these results, analysis of gene expression in ILC2s revealed that over 50% of these genes were upregulated in *Stat4*-deficient ILC2s (Suppl. Figure 3D and Suppl. Table 1), implying that STAT1/STAT4-mediated regulation can operate similarly in both ILC2s and NK cells.

Recent studies have provided evidence that T-bet inhibits a self-reinforcing, type I IFN-mediated autocrine loop in Th1 cells [35]. Given that *Stat4*-deficient iILC2s display aberrant expression of ISGs and a marked increase in IRF7, a key driver of type I IFN production, we hypothesized that STAT4, similarly to T-bet, restrains expression of *Ifna* and/or *Ifnb1* transcripts in ILC2s. Consistent with this hypothesis, *Stat4*-deficient iILC2s exhibited increased levels of *Ifnb1* following IL-25 administration in mice (Fig. 5E).

To assess the role of type I IFNs on the maintenance of the iILC2 pool, we employed an in vitro system using FACS-sorted KLRG1^hi^ ILC2 isolated from the lungs of *Rag2*^*−/−*^ and *Rag2*^*−/−*^*Stat4*^*−/−*^ mice treated with IL-25. Cells were cultured with IL-25 for 24 h in presence of IFN-β or a blocking antibody against the IFN-α/β receptor (IFNAR). As previously reported [22], the number of *Stat4*-sufficient ILC2s rapidly declined upon treatment with IFN-β, while IFNAR blockade had no significant effect (Fig. 5F, left panel). In contrast, IFNAR blockade increased the number of *Stat4*-deficient ILC2 compared with cells treated with IL-25 alone (Fig. 5F, right panel), suggesting a previously unrecognized role for STAT4 in restraining an autocrine type I IFN circuit in iILC2s.

Collectively, these data indicate that, in addition to regulating iILC2 fitness, STAT4 modulates IFN responses and prevents aberrant autocrine type I IFN signaling in iILC2s.

## Discussion

In the classical monolithic model of T cell differentiation, one STAT-one fate, the expression of the SRTF STAT4 is traditionally associated with Th1 lineage commitment and inhibition of the Th2 fate [32, 36, 37]. However, accumulating evidence has challenged this rigid view and STAT4 expression, for instance, has also been reported in multiple differentiated Th subsets, where it functions as a key driver of plasticity toward IFN-γ-producing phenotypes [38–40]. Consistent with this broader role, innate lymphocytes can also be plastic and acquire phenotypes associated with alternative fates [41–43]. In human ILC2s, IL-1β induces the expression of *TBX21*, *CXCR3*, *IL12RB*1, and *IL12RB2*, promoting generation of cells capable of producing high levels of IFN-γ in response to IL-12 stimulation [29, 30]. During viral infection or in settings of chronic obstructive pulmonary disease (COPD), the conversion of ILC2s into ILC1-like cells can be triggered by IL-12 and IL-18, this cell state is accompanied by a downregulation of GATA-3 [31]. Our findings showing STAT4 expression in activated ILC2s reveal an additional layer of complexity. Contrary to expectations, STAT4 does not inhibit type 2 functions nor enhance the plasticity of ILC2s toward a type 1 phenotype, instead, this TF supports the iILC2 pool. Indeed, acute IL-25 stimulation upregulates STAT4 expression in ILC2s and coincides with the appearance of iILC2s in the lung. Under these conditions, STAT4 expression is not associated with upregulation of T-bet or IFN-γ expression, which are the hallmark of the type 1 response.

The IFN-β produced in type 1/type 2 mixed inflammatory responses can suppress the function of ILC2s and Th2 [44]. Moreover, interferons and IL-27 can generally provide a negative feedback mechanism for ILC2 functions by activating STAT1-dependent programs [21–23]. Unexpectedly, we found that in a typical type 2 model, the overall deletion of *Stat4*, which is conventionally considered the major inducer of IFN-γ, led to impaired iILC2 numbers in the lung and upregulation of transcriptome related to type I IFNs. A possible explanation for this effect comes from parallel findings in NK cells, where IFNs can induce STAT1-driven transcriptional programs that are modulated by STAT4 [34]. During viral infection, loss of STAT4 in NK cells results in the upregulation of a subset of STAT1-regulated genes, many of which overlap with the genes upregulated in *Stat4*-deficient ILC2s following IL-25 stimulation. These findings suggest shared regulatory networks between ILC2s and NK cells. Moreover, loss of T-bet in Th1 cells can also lead to upregulation of IFN-transcriptome downstream of IFN-γ stimulation or during infection [35]. Analogously to the function of T-bet in Th1, we found that STAT4 can inhibit a type I IFN-mediated autocrine loop that controls iILC2 fitness, in vitro. Whether this effect in Th1 cells depends solely on T-bet or is additionally modulated by differential STAT4 expression remains to be determined. Likewise, it remains to be determined whether Th2 cells exhibit STAT4-dependent regulatory mechanisms comparable to those observed in ILC2. Supporting this possibility, we observed elevated DNA-accessibility and *Stat4* transcripts in lung Th2 cells from *N. brasiliensis* infected mice.

Under strongly polarized type 2 conditions, STAT4-dependent tolerance to IFNs in ILC2s may exert both protective and pathogenic effects in a context-dependent manner. For instance, during helminth infection, STAT4 expression may sustain ILC2 fitness through a cell-autonomous mechanism that preserves type 2 effector functions and facilitates parasite control. Conversely, in allergic contexts such as asthma, STAT4 expression may exacerbate pathology by promoting the activity of iILC2s. Our findings may also help explain why, in certain cases of virus-induced asthma exacerbation, type 2 effector cell activity still remains pronounced, contributing to increased clinical severity and prolonged viral persistence [45–48]. While our study primarily focused on elucidating the role of STAT4 in regulating ILC2 functions, further investigation using conditional *Stat4* knockout mice, in which the gene is selectively ablated in ILC2s, will be essential to distinguish the protective versus pathogenic contributions of this transcription factor within type 2 immunity. Overall, our findings establish a conceptual framework for exploring the context-dependent role of STAT4 in disease settings such as helminth infection and, notably, asthma conditions in which type 2 inflammation frequently coexists with viral challenges.

## Methods

### Mouse models

C57BL/6j and *Rag2*^*−/−*^ (Jackson mouse stock number 008449) mice were purchased by Charles River. *Stat4*^*−/−*^ mice were a kind gift from Professor M. Kaplan (generated as described previously) [32]. *Rag2*^*−/−*^*Stat4*^*−/−*^ mice were obtained by crossing *Rag2*^*−/−*^ and *Stat4*^*−/−*^ mice. All the experiments were performed using littermates and 6–12 weeks old mice. Experiments were performed using age-matched male and female mice. For sample size, see corresponding Figure legends.

### Cell isolation, flow cytometry and cell sorting

Cells from lung and intestinal lamina propria were isolated after incubation in RPMI containing 0.5 mg/mL DNase I and 0.25 mg/mL Liberase TL (Roche) and purification with 40% Percoll (Cytiva), as described before [49]. Flow cytometry analysis was performed on a LSR Fortessa (Becton Dickinson). Cell sorting was performed using FACSAria III (Becton Dickinson) equipped with 488, 561, Near UV375 and 633 nm laser and FACSDiva software (BD Biosciences version 6.1.3). To reduce stress, cells were isolated in gentle FACS-sorting conditions using a ceramic nozzle of size 100 μm, a low sheath pressure of 19.84 pound-force per square inch (psi) that maintains the sample pressure at 18.96 psi and an acquisition rate of maximum 3,000 events/s. FACS-sorted cells were confirmed to be higher than 95% pure with post-sort analysis. Dead cells were excluded by using Fixable Viability Stain 780 (Becton Dickinson), Zombie NIR™ Fixable Viability Kit (Biolegend), or Zombie Green™ Fixable Viability Kit (Biolegend). Flow cytometry data were analyzed using FlowJo_v10.8.1 (Beckton Dickinson).

### Antibodies and clones

Cell surface staining was performed using the following anti-mouse antibodies: anti-CD45.2 (clone 104) BUV395; CD45.1 (clone A20) Pacific Blue; CD3ε (clone 145-2C11) FITC or BV605; CD19 (clone 1D3) FITC or BV605; NKp46 (CD335, clone 29A1.4) APC; NK1.1 (clone PK136) BV650; KLRG1 (clone 2F1) PerCP-Cy5.5 or BV786; CD127 (clone A7R34) PE-Cy7 or BUV737; IL-33R (clone U29-93) PE or BV510; PD-1 (clone 29F.1A12) APC; IL-25R (clone FAB104102A) APC. Mouse BD Fc Block™ (CD16/CD32, 2.4G2) was used to limit non-specific labeling. For intracellular staining, cells were fixed and permeabilized by using; FoxP3/Transcription Factor Staining Buffer Set (eBioscience) for TFs, phenotypic analysis, and cytokines evaluation; or methanol for evaluation of total- and phospho-STAT4. Intracellular staining was performed using the following anti-mouse antibodies: IFN-γ (XMG1.2) APC-H7, IL-5 (TRFK5) APC, IL-13 (eBio13A) PE, IL-17 (eBio17B7) EF506, CTLA-4 (clone UC10-4B9) PE-Cy7, GATA-3 (L50-823) AF488 and eFluor660, Rorγt (Q21-559, B2D) PECF-594 and PE-Cy7, T-bet (eBio4B10, 4B10) APC or BV785, Ki-67 (SolA15). tSTAT4, pSTAT4, and pSTAT1 were stained after methanol fixation, using the following antibodies: anti- polyclonal total STAT4 (Invitrogen), followed by PE or AF647 Donkey anti-rabbit IgG (Poly4064) labeling, STAT4 (pY693) (38/p-Stat4) PECF-594, STAT1 (pY701) AF647.

### DSS induced colitis

Experimental colitis was induced using a well-established protocol based on DSS administration [50]. DSS (MP Biomedicals) was dissolved in drinking water and administered ad libitum for 7 days. Disease progression was monitored daily by assessing body weight, fecal consistency, presence of fecal blood, and rectal bleeding to calculate the Disease Activity Index (DAI) [51].

### Ex vivo assays

Cells were cultured in RPMI medium with 10% (vol/vol) FCS, 2 mM glutamine, 100 IU/ml of penicillin, 0.1 mg/ml, of streptomycin and 20 mM HEPES buffer, pH 7.2–7.5, 1 mM sodium pyruvate, nonessential amino acids (all from ThermoFisher Scientific). Cytokines were used at the following concentrations: IL-7 (25 ng/mL, Peprotech), IL-25 (50 ng/mL, R&D systems), IL-33 (50 ng/mL, PeproTech), IL-12 (100 ng/ml, Peprotech), IFN-β (10,000 UI/mL, PBL assay), according to the experimental design. Anti-IFNAR (anti-mouse IFNα/β Receptor 1, clone MAR1-5A3) was used at 10μg/mL. For the evaluation of cytokine expression, leukocytes were stimulated using the PMA/Ionomycin-based cell stimulation cocktail (eBioscience), GolgiStop (Becton Dickinson) and GolgiPlug (Becton Dickinson) were added according to the manufacturer instructions. Cell counts were performed by flow cytometry using precision counts beads (Biolegend).

### Bone marrow chimeras

Lineage-positive BM cells were depleted from *Stat4*^+*/*+^ (CD45.1^+^CD45.2^+^) and *Stat4*^*−/−*^ (CD45.2^+^) mice by using the Lineage Cell Depletion kit (Miltenyi Biotec). Cells were washed and resuspended in saline solution, then *Stat4*^+*/*+^ and *Stat4*^*−/−*^ cells were mixed in a 1.1 ratio and intravenously transferred to age- and sex-matched congenic CD45.1^+^ hosts previously lethally irradiated (day-1) at 950 cGy. Experiments were performed 8–12 weeks later.

### Quantitative real-time PCR

Total RNA from sorted KLRG1^hi^ ILC2s was extracted with single cell RNA purification kit (Norgen) according to the manufacturer’s protocol. RNA quantitation and quality were performed using a NanoDrop spectrophotometer (ThermoFisher Scientific). cDNA was carried out in a 25μL reaction volume with 1 g of total RNA according to the manufacturer’s protocol for reverse transcriptase (Promega). cDNAs were amplified in duplicate (sequence for oligonucleotides used is reported in Table 1). *Gapdh* was used as an endogenous reference gene to normalize the expression of *Ifna* isoforms (1, 2, 5, 6 and 7) and *Ifnb1.* All PCR reactions were performed using PowerSYBR Green PCR master mix (ThermoScientific) on an ABI Prism 7900 Sequence Detection System (Applied Biosystems).

### Bulk RNA-seq and transcriptomic analysis

For bulk RNA-seq experiments 30–50,000 ILC2s were FACS-sorted as L/D^−^CD3γ^−^CD19^−^NK1.1^−^KLRG1^+^ cells from the lungs of IL-25-treated (3 days) *Rag2*^*−/−*^ and *Rag2*^*−/−*^*Stat4*^*−/−*^ mice. Four replicates for *Stat4*-sufficient and -deficient ILC2s were generated. RNA was isolated by single cell purification kit (Norgen, #51800) using RNase-Free DNase (Norgen #25710). Libraries were sequenced on an Illumina Novaseq 6000 (by Xenovea Ltd., Szeged, Hungary). The resulting FASTQ files were preprocessed with *Trimmomatic* (v0.39) for adapter trimming and low-quality filtering of the reads [52]. Filtered sequence reads were aligned to reference GRCm38 mouse transcriptome (mm10) with *HISAT2* aligner (v2.2.1) [53] and raw counts were obtained with *FeatureCounts* (subread v2.0.1) [54]. GENCODE Gene Set version M27 was used for gene annotation. Transcripts were merged to genes using R (v 4.4.2 (2024–10–31) – "Pile of Leaves") and *biomaRt* (v2.62.1) [55]. Gene-level normalization and differential expression analysis were performed with the R package *DESeq2* (v1.46.0) [56]. We analyzed genes with a total raw count above 10 in at least three samples. We considered DEGs with |Log_2_ FC|> 1 and an adjusted *p*-value < 0.05. GSEA analysis was performed utilizing the entire MSigDB library for *Mus musculus* using GSEA software (v4.3.3) from the UC San Diego and Broad institute [57, 58].

### Single cell RNA-seq analysis

Publicly available scRNA-seq data of murine lung ILC2s from *N. brasiliensis* infected mice were retrieved using the accession code GSE131996. Only ILC2 clusters were analyzed, raw counts were processed using R and the package *Seurat* (v5.3.0) [59] according to standard pipeline. Gene markers were identified using the “*FindAllMarkers*” function with the following parameters (min.pct = 0.2, logfc.threshold = 0.2, min.diff.pct = 0). For the visualization of the Umap and gene expression, we utilized the R package *SCpubr* (v 2.0.2) [60].

### Statistical analysis

Detailed information on the number of mice analyzed and the statistical methods applied is provided in the figure legends. All experiments were independently repeated at least twice to ensure reproducibility. Comparisons between two groups were analyzed using an unpaired or paired Student’s *t*-test, according to the experimental groups, whereas one-way ANOVA followed by Tukey’s multiple comparisons test was used for experiments involving multiple groups. Statistical analyses were performed using GraphPad Prism version 8.0.2 (GraphPad Software). Differences were considered statistically significant at *P* < 0.05 and are indicated by asterisks in the figures.

## Supplementary Information

Below is the link to the electronic supplementary material.

Supplementary file1 (DOCX 365 KB)

## Data Availability

The bulk RNA-seq dataset generated during the current study has been deposited in the GEO under accession number GSE305160.
