# Supplementary material for: STAT4 drives optimal expansion and transcriptional repression of type I interferon pathway in inflammatory ILC2
Source: Cell Mol Life Sci. 2026 Mar 26;83(1):193. doi: 10.1007/s00018-026-06157-6 (PMC13049133; doi:10.1007/s00018-026-06157-6)
Supplement: Supplementary file 1 — Supplementary file1 (DOCX 365 KB) [file 18_2026_6157_MOESM1_ESM.docx]

**Supplementary Figure Legends**

**Supplementary Figure 1. scRNA-seq analysis of lung ILC2s from mice infected with *N. brasiliensis***

**(A)** Umap representation obtained from scRNA-seq data of lung ILC2s isolated from mice infected with *N. brasiliensis*. Two clusters of nILC2s, nILC2a and nILC2b, and a cluster of iILC2s were identified (see **Suppl. Table 1**).

**(B)** Dot plot representing scaled values of expression of selected markers in distinct ILC2 clusters, during infection.

**
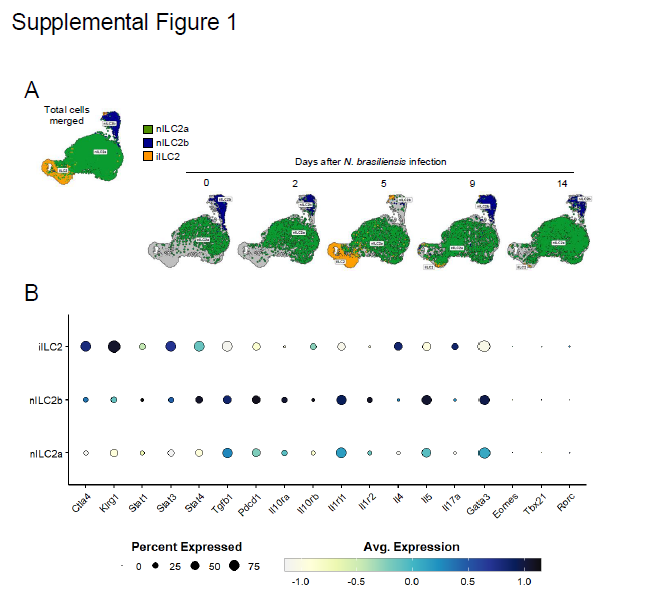
**

**Supplementary Figure 2. Flow cytometry gating strategies and controls**

**(A)** Gating strategy for the identification of ILC2s from tissues. Live/dead(L/D)^-^CD45^+^CD3ε^-^CD19^-^NK1.1^-^Rorγt^-^ cells were gated and further dissected for the expression of GATA-3 to define ILC2s. Total NK1.1^+^ cells, consisting of NK cells and ILC1, were also included in the analysis. nILC2s and iILC2s were discriminated based on KLRG1, IL-33R or CD127 expression in IL-25 treated mice.

**(B)** Representative flow cytometry histogram plots depict STAT4 expression in ILC2s isolated from small intestine lamina propria (SILP), lung, mesenteric lymph nodes (Mes LN) or large intestine lamina propria (LILP) of untreated and IL-33-treated mice.

**(C)** Representative flow cytometry histogram plots depict expression of IFN-γ, IL-5, IL-13 and IL-17 in lung ILC2s from IL-25 treated mice upon ex vivo restimulation with PMA/Ionomycin for three hours.

**(D)** Ratio of total CD45.2^+^ (*Stat4^+/+^*) and CD45.1^+^CD45.2^+^ (*Stat4^-/-^*) cells from the lung of mice treated with IL-25. Representative flow cytometry (left panel) and a representative experiment (right panel) from two independent experiments (*n=* 3) are shown. For statistical analysis, unpaired Student’s t test was used.


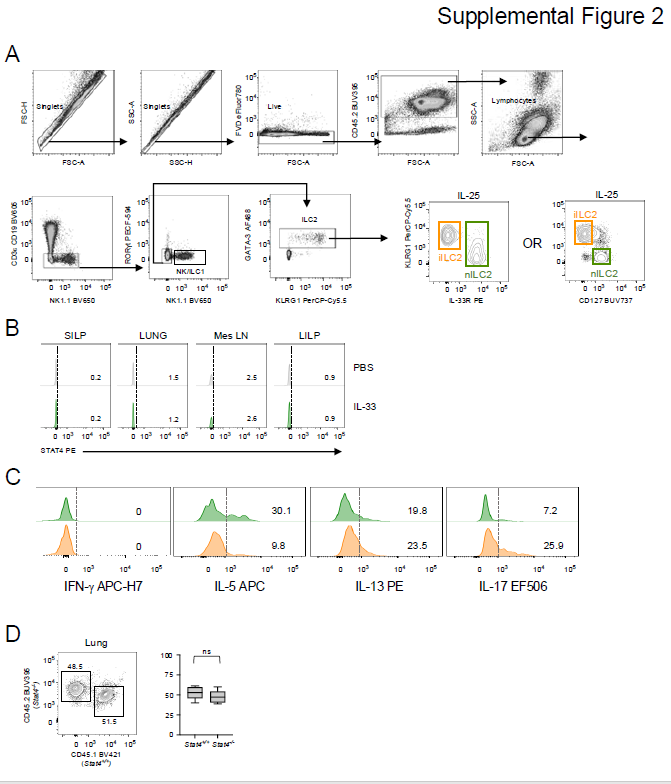


**Supplementary Figure 3. FACS sorting strategy and transcriptomic analysis**

**(A**) Gating strategy for FACS-sorted KLRG1^hi^ ILC2s isolated from the lung of IL-25 treated *Rag2^-/-^* and *Rag2^-/-^Stat4^-/-^* mice, and expression of markers for iILC2 identity.

**(B)** Expression of selected genes in ILC2 from *Rag2^-/-^* and *Rag2^-/-^Stat4^-/-^* mice from bulk RNA-seq.

**(C)** Flow cytometry histogram plot shows expression of STAT4 in iILC2s after ex vivo stimulation with IL-25, IL-12, and IFN-β alone, for 30 minutes. A representative experiment (*n=*3) of two independent experiments performed is shown.

**(D)** Shared STAT4/STAT1 targets in NK cells were plotted in ILC2s from *Rag2^-/-^* and *Rag2^-/-^Stat4^-/-^* mice.


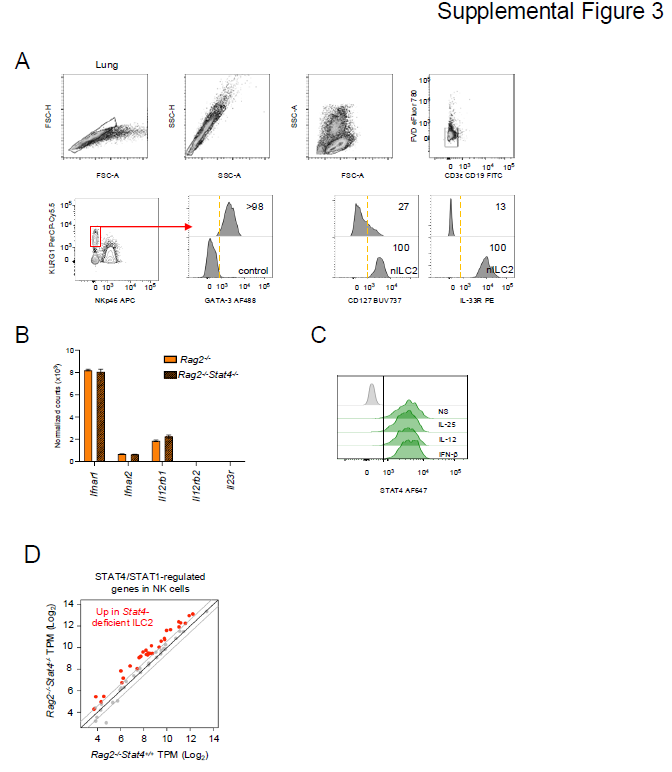
**Supplementary Table 1.**

**Table relative to Fig. 1C and D.** Gene markers defining iILC2 and nILC2 at day five after *N. brasiliensis* infection.

|  | ***scRNA-seq / ILC2 at day 5 post N.brasiliensis infection (top 100)*** | | | | | | |
| --- | --- | --- | --- | --- | --- | --- | --- |
| p_val | avg_log2FC | pct.1 | pct.2 | p_val_adj | cluster | gene |  |
| 0 | 4.627161607 | 0.876 | 0.102 | 0 | nILC2a | *Ramp1* |  |
| 0 | 4.477506246 | 0.824 | 0.176 | 0 | nILC2a | *Fosb* |  |
| 0 | 3.082419992 | 0.951 | 0.448 | 0 | nILC2a | *S100a4* |  |
| 0 | 3.086230162 | 0.977 | 0.594 | 0 | nILC2a | *Ctla2a* |  |
| 0 | 0.719931926 | 1 | 0.999 | 0 | nILC2a | *Rps29* |  |
| 0 | 1.568955506 | 0.999 | 0.999 | 0 | nILC2a | *Malat1* |  |
| 2.02E-302 | 2.665824589 | 0.915 | 0.607 | 3.11E-298 | nILC2a | *Zfp36l1* |  |
| 6.91E-298 | 0.700366242 | 1 | 0.999 | 1.07E-293 | nILC2a | *Tpt1* |  |
| 6.50E-295 | 0.739669052 | 1 | 0.997 | 1.00E-290 | nILC2a | *Rps24* |  |
| 7.20E-293 | 0.759671765 | 1 | 0.999 | 1.11E-288 | nILC2a | *Rpl37a* |  |
| 7.87E-280 | 4.047275069 | 0.744 | 0.156 | 1.21E-275 | nILC2a | *Ltb* |  |
| 1.61E-278 | 0.71312688 | 1 | 0.998 | 2.49E-274 | nILC2a | *Rpl21* |  |
| 4.32E-277 | 0.873145247 | 1 | 0.999 | 6.66E-273 | nILC2a | *Rps27* |  |
| 6.49E-273 | 2.142281688 | 0.973 | 0.902 | 1.00E-268 | nILC2a | *Junb* |  |
| 8.43E-272 | 1.417080327 | 1 | 0.981 | 1.30E-267 | nILC2a | *Fth1* |  |
| 1.55E-264 | 0.714277414 | 0.999 | 0.996 | 2.39E-260 | nILC2a | *Rps7* |  |
| 2.49E-262 | 0.689516455 | 0.999 | 0.998 | 3.84E-258 | nILC2a | *Rpl35a* |  |
| 1.33E-261 | 1.365826405 | 0.984 | 0.957 | 2.04E-257 | nILC2a | *H2-K1* |  |
| 1.29E-255 | 4.773166228 | 0.658 | 0.073 | 1.98E-251 | nILC2a | *Ccr2* |  |
| 1.49E-255 | 0.67932415 | 1 | 0.999 | 2.30E-251 | nILC2a | *Rps14* |  |
| 1.38E-254 | 3.555603009 | 0.73 | 0.193 | 2.12E-250 | nILC2a | *Cd81* |  |
| 4.92E-254 | 0.68014755 | 1 | 0.999 | 7.59E-250 | nILC2a | *Rps9* |  |
| 9.26E-240 | 1.432054303 | 0.986 | 0.969 | 1.43E-235 | nILC2a | *Btg1* |  |
| 5.18E-231 | 1.701691533 | 0.97 | 0.915 | 7.99E-227 | nILC2a | *Furin* |  |
| 2.94E-230 | 0.603754315 | 1 | 0.997 | 4.53E-226 | nILC2a | *Rps15a* |  |
| 7.46E-230 | 2.744723742 | 0.794 | 0.396 | 1.15E-225 | nILC2a | *Tmem176b* |  |
| 1.80E-214 | 3.948570126 | 0.627 | 0.108 | 2.78E-210 | nILC2a | *Ccdc184* |  |
| 2.14E-214 | 0.590501279 | 1 | 0.999 | 3.30E-210 | nILC2a | *Rpl9* |  |
| 1.38E-210 | 3.528637705 | 0.749 | 0.363 | 2.12E-206 | nILC2a | *Cxcl2* |  |
| 7.02E-210 | 2.782865643 | 0.754 | 0.373 | 1.08E-205 | nILC2a | *Capg* |  |
| 1.91E-208 | 3.99764202 | 0.658 | 0.182 | 2.94E-204 | nILC2a | *Ccl1* |  |
| 9.28E-206 | 3.81186562 | 0.728 | 0.294 | 1.43E-201 | nILC2a | *Fos* |  |
| 1.65E-202 | 3.924309045 | 0.619 | 0.121 | 2.55E-198 | nILC2a | *Csf2* |  |
| 1.17E-201 | 1.620287853 | 0.898 | 0.799 | 1.80E-197 | nILC2a | *Cd82* |  |
| 4.08E-197 | 0.575883696 | 0.999 | 0.994 | 6.29E-193 | nILC2a | *Rpl38* |  |
| 1.13E-195 | 0.606972922 | 1 | 0.995 | 1.74E-191 | nILC2a | *Rpl17* |  |
| 4.46E-190 | 2.138871741 | 0.784 | 0.383 | 6.88E-186 | nILC2a | *Il1rl1* |  |
| 2.72E-187 | 2.51345366 | 0.756 | 0.329 | 4.19E-183 | nILC2a | *Arg1* |  |
| 3.81E-187 | 0.576323734 | 0.999 | 1 | 5.88E-183 | nILC2a | *Rps15* |  |
| 1.03E-185 | 0.58371156 | 1 | 0.996 | 1.59E-181 | nILC2a | *Rpl34* |  |
| 1.34E-184 | 2.187195789 | 0.91 | 0.788 | 2.06E-180 | nILC2a | *Gadd45b* |  |
| 1.12E-183 | 2.300017052 | 0.762 | 0.439 | 1.73E-179 | nILC2a | *Tmem176a* |  |
| 1.56E-178 | 1.848871988 | 0.841 | 0.609 | 2.40E-174 | nILC2a | *Bhlhe40* |  |
| 2.56E-175 | 0.505697794 | 1 | 0.999 | 3.94E-171 | nILC2a | *Rplp1* |  |
| 4.72E-174 | 2.565787873 | 0.743 | 0.413 | 7.28E-170 | nILC2a | *Hs3st1* |  |
| 1.02E-173 | 0.734484195 | 1 | 1 | 1.57E-169 | nILC2a | *B2m* |  |
| 4.83E-173 | 0.854243127 | 0.992 | 0.988 | 7.45E-169 | nILC2a | *Ly6e* |  |
| 2.87E-172 | 0.503886186 | 1 | 0.997 | 4.43E-168 | nILC2a | *Rpl23* |  |
| 5.79E-169 | 3.470193019 | 0.714 | 0.373 | 8.93E-165 | nILC2a | *Jun* |  |
| 1.73E-166 | 0.466362497 | 0.999 | 0.999 | 2.66E-162 | nILC2a | *Rpl19* |  |
| 1.06E-164 | 0.468697153 | 1 | 1 | 1.63E-160 | nILC2a | *Rpl13* |  |
| 2.40E-160 | 3.178038053 | 0.612 | 0.204 | 3.69E-156 | nILC2a | *Cdkn1a* |  |
| 6.79E-156 | 0.481147355 | 1 | 0.997 | 1.05E-151 | nILC2a | *Rpl23a* |  |
| 3.32E-154 | 0.483551607 | 1 | 0.999 | 5.12E-150 | nILC2a | *Rps19* |  |
| 1.07E-152 | 1.320426933 | 0.877 | 0.788 | 1.64E-148 | nILC2a | *Ptpn18* |  |
| 1.92E-152 | 0.530479158 | 1 | 0.997 | 2.96E-148 | nILC2a | *Rpl37* |  |
| 3.16E-151 | 0.645492681 | 0.999 | 0.999 | 4.87E-147 | nILC2a | *H2-D1* |  |
| 1.52E-148 | 3.406711826 | 0.577 | 0.183 | 2.34E-144 | nILC2a | *Klf4* |  |
| 9.75E-145 | 0.942373698 | 0.978 | 0.945 | 1.50E-140 | nILC2a | *Rps27rt* |  |
| 2.10E-144 | 4.102957146 | 0.457 | 0.055 | 3.24E-140 | nILC2a | *Sdc4* |  |
| 2.33E-144 | 4.774501393 | 0.426 | 0.032 | 3.59E-140 | nILC2a | *Lpcat2* |  |
| 2.81E-143 | 1.731834936 | 0.909 | 0.772 | 4.34E-139 | nILC2a | *Rgs2* |  |
| 2.11E-141 | 0.941092288 | 0.96 | 0.921 | 3.25E-137 | nILC2a | *Xist* |  |
| 1.42E-140 | 1.629151872 | 0.858 | 0.602 | 2.18E-136 | nILC2a | *Areg* |  |
| 2.43E-140 | 1.891829565 | 0.758 | 0.519 | 3.74E-136 | nILC2a | *Tnfaip3* |  |
| 5.09E-136 | 0.624571313 | 0.999 | 1 | 7.84E-132 | nILC2a | *Ubb* |  |
| 3.50E-135 | 0.946608882 | 0.943 | 0.924 | 5.39E-131 | nILC2a | *Pfdn5* |  |
| 1.47E-131 | 0.428938368 | 1 | 0.999 | 2.26E-127 | nILC2a | *Rps4x* |  |
| 2.20E-131 | 3.53141168 | 0.459 | 0.076 | 3.39E-127 | nILC2a | *Stab2* |  |
| 3.78E-131 | 1.899130196 | 0.777 | 0.575 | 5.83E-127 | nILC2a | *Ier5* |  |
| 4.84E-131 | 1.407221894 | 0.941 | 0.83 | 7.46E-127 | nILC2a | *Nfkbia* |  |
| 1.79E-127 | 1.665962085 | 0.745 | 0.55 | 2.76E-123 | nILC2a | *Slc3a2* |  |
| 6.66E-127 | 2.64591007 | 0.635 | 0.33 | 1.03E-122 | nILC2a | *Ppp1r15a* |  |
| 1.01E-126 | 0.443474969 | 1 | 0.995 | 1.56E-122 | nILC2a | *Rps13* |  |
| 7.11E-126 | 0.462956184 | 0.999 | 0.995 | 1.10E-121 | nILC2a | *Rpl26* |  |
| 5.73E-124 | 0.430154422 | 1 | 0.997 | 8.84E-120 | nILC2a | *Rpl39* |  |
| 3.79E-122 | 0.472506426 | 1 | 0.996 | 5.84E-118 | nILC2a | *Rpl36* |  |
| 1.15E-120 | 2.042288431 | 0.654 | 0.358 | 1.77E-116 | nILC2a | *Cish* |  |
| 3.79E-120 | 2.067825764 | 0.65 | 0.33 | 5.83E-116 | nILC2a | *Il2ra* |  |
| 9.34E-119 | 0.436683377 | 0.999 | 0.996 | 1.44E-114 | nILC2a | *Rps28* |  |
| 9.87E-118 | 3.131466713 | 0.544 | 0.203 | 1.52E-113 | nILC2a | *Rgcc* |  |
| 1.65E-115 | 3.439772101 | 0.451 | 0.104 | 2.55E-111 | nILC2a | *Nfkbid* |  |
| 2.11E-114 | 1.950731325 | 0.687 | 0.457 | 3.25E-110 | nILC2a | *Fosl2* |  |
| 9.45E-114 | 3.659071143 | 0.408 | 0.065 | 1.46E-109 | nILC2a | *Camk2n1* |  |
| 1.49E-111 | 3.92775717 | 0.411 | 0.075 | 2.30E-107 | nILC2a | *Atf3* |  |
| 1.09E-110 | 1.457361802 | 0.786 | 0.639 | 1.68E-106 | nILC2a | *H2-Q7* |  |
| 3.12E-110 | 3.302905241 | 0.431 | 0.094 | 4.81E-106 | nILC2a | *Inpp4b* |  |
| 1.13E-109 | 1.679579795 | 0.69 | 0.441 | 1.74E-105 | nILC2a | *Cd44* |  |
| 6.47E-109 | 1.836018566 | 0.703 | 0.491 | 9.97E-105 | nILC2a | *4930523C07Rik* | |
| 3.93E-108 | 4.113017669 | 0.369 | 0.046 | 6.06E-104 | nILC2a | *Prnp* |  |
| 4.53E-108 | 2.42030269 | 0.554 | 0.248 | 6.99E-104 | nILC2a | *Znrf1* |  |
| 1.97E-106 | 0.374616289 | 1 | 0.999 | 3.03E-102 | nILC2a | *Rps27a* |  |
| 4.44E-105 | 1.322476786 | 0.902 | 0.877 | 6.84E-101 | nILC2a | *Pim1* |  |
| 5.61E-105 | 3.024448566 | 0.49 | 0.172 | 8.65E-101 | nILC2a | *Nr4a2* |  |
| 1.24E-104 | 0.48025354 | 0.997 | 0.999 | 1.92E-100 | nILC2a | *Rpl30* |  |
| 1.33E-104 | 2.237277933 | 0.748 | 0.543 | 2.06E-100 | nILC2a | *Il5* |  |
| 1.83E-103 | 3.823640052 | 0.395 | 0.079 | 2.81E-099 | nILC2a | *Nrgn* |  |
| 2.78E-103 | 0.452381501 | 0.997 | 0.989 | 4.29E-099 | nILC2a | *Rps25* |  |
| 8.53E-101 | 1.075428525 | 0.849 | 0.796 | 1.32E-096 | nILC2a | *Il2rg* |  |
| 2.91E-100 | 0.355320619 | 1 | 1 | 4.49E-096 | nILC2a | *Rpl8* |  |
| 2.26E-012 | 5.954705861 | 0.286 | 0.011 | 3.48E-008 | nILC2b | *Gm43403* |  |
| 2.70E-009 | 6.587298461 | 0.286 | 0.014 | 4.17E-005 | nILC2b | *Cd70* |  |
| 5.32E-007 | 3.292787902 | 0.429 | 0.044 | 0.008193437 | nILC2b | *Adm* |  |
| 1.44E-006 | 4.824606476 | 0.429 | 0.049 | 0.0222599 | nILC2b | *Lcor* |  |
| 2.18E-006 | 4.329575163 | 0.286 | 0.022 | 0.033576093 | nILC2b | *Hbegf* |  |
| 0 | 4.869167471 | 0.875 | 0.084 | 0 | iILC2 | *Nkg7* |  |
| 0 | 5.608898713 | 0.776 | 0.032 | 0 | iILC2 | *Ccr9* |  |
| 0 | 3.749145361 | 0.875 | 0.132 | 0 | iILC2 | *Lgmn* |  |
| 0 | 3.801006652 | 0.834 | 0.092 | 0 | iILC2 | *Gimap4* |  |
| 0 | 3.896964437 | 0.781 | 0.083 | 0 | iILC2 | *Rasgrp2* |  |
| 0 | 5.244284898 | 0.723 | 0.027 | 0 | iILC2 | *Gimap7* |  |
| 0 | 5.108662001 | 0.711 | 0.029 | 0 | iILC2 | *Ms4a4b* |  |
| 0 | 3.865980388 | 0.91 | 0.241 | 0 | iILC2 | *Plac8* |  |
| 0 | 2.644087086 | 0.893 | 0.226 | 0 | iILC2 | *Lgals3* |  |
| 0 | 3.145094695 | 0.863 | 0.199 | 0 | iILC2 | *Cd48* |  |
| 0 | 3.554133422 | 0.766 | 0.114 | 0 | iILC2 | *Ctla4* |  |
| 0 | 4.644361233 | 0.651 | 0.033 | 0 | iILC2 | *Ms4a6b* |  |
| 0 | 2.620643216 | 0.9 | 0.342 | 0 | iILC2 | *Pycard* |  |
| 0 | 2.142613247 | 0.968 | 0.46 | 0 | iILC2 | *Klrg1* |  |
| 0 | 1.23667701 | 0.998 | 0.871 | 0 | iILC2 | *Coro1a* |  |
| 0 | 1.589307115 | 0.999 | 0.913 | 0 | iILC2 | *Lgals1* |  |
| 0 | 1.811708228 | 0.995 | 0.917 | 0 | iILC2 | *S100a10* |  |
| 0 | 6.486917887 | 0.732 | 0.131 | 7.19E-304 | iILC2 | *Gzma* |  |
| 3.33E-302 | 1.648521137 | 0.975 | 0.663 | 5.13E-298 | iILC2 | *Lsp1* |  |
| 2.56E-300 | 1.508786079 | 0.994 | 0.798 | 3.94E-296 | iILC2 | *Itgb7* |  |
| 4.44E-296 | 3.416410437 | 0.721 | 0.104 | 6.84E-292 | iILC2 | *Rilpl2* |  |
| 4.68E-292 | 1.372086632 | 0.996 | 0.974 | 7.22E-288 | iILC2 | *Ftl1* |  |
| 2.38E-291 | 2.33665342 | 0.902 | 0.353 | 3.67E-287 | iILC2 | *Anxa2* |  |
| 6.71E-289 | 2.45636836 | 0.865 | 0.289 | 1.03E-284 | iILC2 | *Cmtm7* |  |
| 1.74E-285 | 1.444085119 | 0.988 | 0.762 | 2.68E-281 | iILC2 | *Selplg* |  |
| 4.15E-281 | 1.086483071 | 1 | 0.996 | 6.40E-277 | iILC2 | *Actg1* |  |
| 3.50E-280 | 4.705990588 | 0.593 | 0.036 | 5.39E-276 | iILC2 | *1500009L16Rik* | |
| 2.08E-275 | 2.349265921 | 0.882 | 0.302 | 3.21E-271 | iILC2 | *Lztfl1* |  |
| 2.00E-274 | 4.347706396 | 0.588 | 0.035 | 3.09E-270 | iILC2 | *Cd79b* |  |
| 1.14E-257 | 4.43325331 | 0.542 | 0.023 | 1.76E-253 | iILC2 | *Cdc25b* |  |
| 1.08E-252 | 1.755642336 | 0.928 | 0.432 | 1.66E-248 | iILC2 | *Tpm4* |  |
| 3.33E-247 | 1.675332084 | 0.981 | 0.628 | 5.14E-243 | iILC2 | *Gm2a* |  |
| 1.29E-245 | 2.639866478 | 0.77 | 0.196 | 1.98E-241 | iILC2 | *Rhoc* |  |
| 1.31E-242 | 1.107117714 | 0.989 | 0.802 | 2.02E-238 | iILC2 | *Cdc42* |  |
| 4.69E-242 | 1.236852581 | 0.998 | 0.925 | 7.23E-238 | iILC2 | *Crip1* |  |
| 5.04E-238 | 1.298911293 | 0.974 | 0.805 | 7.78E-234 | iILC2 | *Anp32a* |  |
| 2.34E-235 | 1.148271576 | 0.996 | 0.869 | 3.60E-231 | iILC2 | *Emb* |  |
| 3.91E-230 | 2.484088706 | 0.729 | 0.184 | 6.03E-226 | iILC2 | *Stmn1* |  |
| 8.56E-230 | 1.328983775 | 0.971 | 0.665 | 1.32E-225 | iILC2 | *Cd47* |  |
| 2.22E-229 | 2.029883345 | 0.84 | 0.263 | 3.42E-225 | iILC2 | *Itga4* |  |
| 4.78E-229 | 4.011386485 | 0.515 | 0.031 | 7.36E-225 | iILC2 | *Mras* |  |
| 2.06E-226 | 2.426488108 | 0.719 | 0.169 | 3.18E-222 | iILC2 | *Lmnb1* |  |
| 1.98E-225 | 6.199784436 | 0.45 | 0.006 | 3.06E-221 | iILC2 | *Hsbp1l1* |  |
| 1.73E-223 | 4.768659158 | 0.49 | 0.026 | 2.67E-219 | iILC2 | *Ggt1* |  |
| 1.03E-222 | 2.332400559 | 0.729 | 0.163 | 1.59E-218 | iILC2 | *Ctsa* |  |
| 1.32E-222 | 2.846134749 | 0.665 | 0.127 | 2.03E-218 | iILC2 | *S1pr4* |  |
| 1.37E-220 | 1.090017769 | 0.98 | 0.83 | 2.11E-216 | iILC2 | *Arhgdib* |  |
| 2.49E-220 | 1.397156763 | 0.953 | 0.596 | 3.84E-216 | iILC2 | *Gmfg* |  |
| 2.51E-220 | 2.282422044 | 0.774 | 0.234 | 3.87E-216 | iILC2 | *Atp1b3* |  |
| 1.12E-219 | 4.260601361 | 0.488 | 0.025 | 1.72E-215 | iILC2 | *Dhrs3* |  |
| 4.99E-219 | 3.713223787 | 0.516 | 0.039 | 7.70E-215 | iILC2 | *Sla2* |  |
| 2.70E-218 | 2.326076166 | 0.759 | 0.215 | 4.16E-214 | iILC2 | *Batf* |  |
| 1.64E-217 | 2.284703649 | 0.769 | 0.213 | 2.52E-213 | iILC2 | *Ikzf2* |  |
| 1.92E-211 | 2.597806986 | 0.635 | 0.106 | 2.96E-207 | iILC2 | *Irak3* |  |
| 2.07E-210 | 3.262600367 | 0.531 | 0.051 | 3.19E-206 | iILC2 | *Dus2* |  |
| 1.97E-209 | 1.829557107 | 0.86 | 0.344 | 3.04E-205 | iILC2 | *Glrx* |  |
| 1.06E-206 | 2.349902821 | 0.689 | 0.152 | 1.63E-202 | iILC2 | *Pon2* |  |
| 2.08E-206 | 1.160836102 | 0.967 | 0.729 | 3.21E-202 | iILC2 | *Cox5b* |  |
| 1.24E-205 | 3.465751225 | 0.499 | 0.041 | 1.91E-201 | iILC2 | *Ube2c* |  |
| 2.50E-204 | 3.025354771 | 0.569 | 0.078 | 3.86E-200 | iILC2 | *Pou2f2* |  |
| 2.90E-201 | 0.869638546 | 0.993 | 0.898 | 4.47E-197 | iILC2 | *Arpc1b* |  |
| 1.38E-198 | 1.021443989 | 0.978 | 0.819 | 2.13E-194 | iILC2 | *Ppp1ca* |  |
| 2.85E-198 | 1.983119121 | 0.791 | 0.288 | 4.39E-194 | iILC2 | *Dnajc15* |  |
| 9.01E-198 | 3.629018276 | 0.525 | 0.069 | 1.39E-193 | iILC2 | *2810417H13Rik* | |
| 5.65E-197 | 2.737157418 | 0.57 | 0.086 | 8.72E-193 | iILC2 | *Cenpa* |  |
| 2.55E-196 | 1.486909621 | 0.909 | 0.445 | 3.93E-192 | iILC2 | *Supt4a* |  |
| 6.53E-196 | 1.80177229 | 0.841 | 0.351 | 1.01E-191 | iILC2 | *Sms* |  |
| 6.68E-195 | 4.681870803 | 0.425 | 0.016 | 1.03E-190 | iILC2 | *Rom1* |  |
| 4.96E-190 | 1.233207825 | 0.954 | 0.606 | 7.64E-186 | iILC2 | *Cytip* |  |
| 7.59E-190 | 5.276523362 | 0.396 | 0.008 | 1.17E-185 | iILC2 | *Myl4* |  |
| 2.91E-188 | 3.653112157 | 0.487 | 0.054 | 4.48E-184 | iILC2 | *Birc5* |  |
| 6.47E-187 | 3.304574581 | 0.518 | 0.071 | 9.97E-183 | iILC2 | *Ifi27l2a* |  |
| 5.52E-185 | 4.249028274 | 0.442 | 0.032 | 8.50E-181 | iILC2 | *Tppp3* |  |
| 1.37E-183 | 1.578694711 | 0.908 | 0.434 | 2.11E-179 | iILC2 | *Gpr171* |  |
| 2.43E-181 | 2.794549412 | 0.519 | 0.066 | 3.74E-177 | iILC2 | *Hip1r* |  |
| 1.71E-180 | 2.995417282 | 0.5 | 0.062 | 2.63E-176 | iILC2 | *Ctsh* |  |
| 2.63E-180 | 2.064692608 | 0.719 | 0.253 | 4.05E-176 | iILC2 | *Hmgn2* |  |
| 7.81E-180 | 1.651406546 | 0.841 | 0.334 | 1.20E-175 | iILC2 | *Anxa11* |  |
| 1.31E-179 | 1.05112655 | 0.98 | 0.742 | 2.02E-175 | iILC2 | *Rap1b* |  |
| 1.35E-179 | 2.211495628 | 0.632 | 0.14 | 2.09E-175 | iILC2 | *Pigt* |  |
| 8.75E-179 | 2.47823583 | 0.558 | 0.093 | 1.35E-174 | iILC2 | *Hexb* |  |
| 4.22E-178 | 2.657106039 | 0.566 | 0.112 | 6.50E-174 | iILC2 | *Cks1b* |  |
| 1.84E-176 | 1.848344936 | 0.746 | 0.241 | 2.84E-172 | iILC2 | *Myo1g* |  |
| 1.16E-175 | 2.142813319 | 0.628 | 0.137 | 1.79E-171 | iILC2 | *Snx2* |  |
| 1.63E-175 | 3.620567757 | 0.421 | 0.026 | 2.51E-171 | iILC2 | *Rasa3* |  |
| 2.53E-175 | 3.080199786 | 0.449 | 0.038 | 3.89E-171 | iILC2 | *Ccnb2* |  |
| 2.58E-175 | 3.592064588 | 0.427 | 0.029 | 3.98E-171 | iILC2 | *Cdca3* |  |
| 1.99E-174 | 3.566722307 | 0.441 | 0.039 | 3.07E-170 | iILC2 | *Phf11b* |  |
| 3.22E-173 | 2.61101944 | 0.528 | 0.08 | 4.97E-169 | iILC2 | *Otulin* |  |
| 1.63E-172 | 2.035232384 | 0.698 | 0.2 | 2.51E-168 | iILC2 | *Arap2* |  |
| 9.19E-172 | 1.123778076 | 0.958 | 0.681 | 1.42E-167 | iILC2 | *Reep5* |  |
| 2.30E-171 | 1.314505298 | 0.902 | 0.48 | 3.55E-167 | iILC2 | *Clta* |  |
| 4.34E-171 | 2.055100874 | 0.646 | 0.161 | 6.68E-167 | iILC2 | *Rab8a* |  |
| 1.38E-170 | 1.307110375 | 0.968 | 0.775 | 2.13E-166 | iILC2 | *Hmgb1* |  |
| 7.18E-170 | 1.44314334 | 0.857 | 0.382 | 1.11E-165 | iILC2 | *Rbx1* |  |
| 6.05E-169 | 1.508274337 | 0.87 | 0.394 | 9.33E-165 | iILC2 | *Ssr2* |  |
| 2.02E-168 | 2.163469457 | 0.596 | 0.127 | 3.11E-164 | iILC2 | *Hspa14* |  |
| 2.89E-167 | 1.301693306 | 0.91 | 0.49 | 4.46E-163 | iILC2 | *Rps27l* |  |
| 6.46E-167 | 1.684269047 | 0.787 | 0.286 | 9.95E-163 | iILC2 | *Ezr* |  |
| 3.53E-166 | 1.336828371 | 0.924 | 0.568 | 5.44E-162 | iILC2 | *Ostf1* |  |

**Table relative to Fig. 5A.** Full list of DEGs for ILC2 isolated from IL-25-treated *Rag2^-/-^* and *Rag2^-/-^Stat4^-/-^* mice.

| ***Down-regulated genes in Stat4-deficient ILC2*** | | | | |
| --- | --- | --- | --- | --- |
|  |  |  |  |  |
|  | gene | log2FC | padj |  |
|  | *Bpifa1* | -4.48099 | 4.46E-21 |  |
|  | *Ace* | -2.79307 | 5.83E-30 |  |
|  | *Fcgr4* | -2.67755 | 6.31E-06 |  |
|  | *Cxcl15* | -2.60586 | 1.58E-11 |  |
|  | *Tlr13* | -2.39811 | 0.000689 |  |
|  | *Hck* | -2.13837 | 0.000442 |  |
|  | *Cbr2* | -2.06033 | 1.9E-07 |  |
|  | *Bpifb1* | -1.99483 | 0.001901 |  |
|  | *Clec4a1* | -1.97165 | 0.000408 |  |
|  | *Plbd1* | -1.9302 | 3.8E-07 |  |
|  | *Clec4a3* | -1.92533 | 0.000279 |  |
|  | *Klrb1c* | -1.90645 | 0.002249 |  |
|  | *Csf1r* | -1.90272 | 5.01E-15 |  |
|  | *Lyz2* | -1.89364 | 7.66E-34 |  |
|  | *Amd2* | -1.86925 | 7.41E-08 |  |
|  | *Sftpa1* | -1.86447 | 1.59E-21 |  |
|  | *Slc34a2* | -1.85188 | 1.83E-11 |  |
|  | *Lamp3* | -1.73389 | 0.002003 |  |
|  | *Rn18s* | -1.72592 | 0.089702 |  |
|  | *Sftpc* | -1.70678 | 2.71E-43 |  |
|  | *Fcgr2b* | -1.70631 | 6.49E-05 |  |
|  | *Marcks* | -1.67277 | NA |  |
|  | *Sftpd* | -1.63024 | 0.002735 |  |
|  | *Mrc1* | -1.6283 | 0.004753 |  |
|  | *Sirpa* | -1.58101 | 1.56E-06 |  |
|  | *Stat4* | -1.5652 | 0 |  |
|  | *Fgr* | -1.52938 | 0.000213 |  |
|  | *Itgax* | -1.51607 | 8.6E-07 |  |
|  | *Cd300a* | -1.4972 | 0.013807 |  |
|  | *Pld4* | -1.4704 | 5.83E-05 |  |
|  | *Rn45s* | -1.44103 | 0.219578 |  |
|  | *Lars2* | -1.41558 | NA |  |
|  | *Ksr2* | -1.4117 | 0.012269 |  |
|  | *Cyp2f2* | -1.4117 | 0.008393 |  |
|  | *Chi3l1* | -1.39101 | 5.97E-06 |  |
|  | *Sulf2* | -1.34506 | 0.032665 |  |
|  | *Tgm2* | -1.32954 | 7.02E-08 |  |
|  | *Sftpb* | -1.32606 | 1.21E-08 |  |
|  | *H3c13* | -1.31201 | 0.055269 |  |
|  | *Thbs1* | -1.29189 | 1.41E-06 |  |
|  | *H2ac13* | -1.28947 | 0.010339 |  |
|  | *G530011O06Rik* | -1.28693 | 0.00012 |  |
|  | *Cd36* | -1.2782 | 0.000601 |  |
|  | *Cebpa* | -1.25664 | 0.035988 |  |
|  | *Il1b* | -1.25554 | 0.000869 |  |
|  | *Slc3a1* | -1.18159 | 0.093282 |  |
|  | *Mid1* | -1.16895 | 2.15E-12 |  |
|  | *Pirb* | -1.14736 | 0.009069 |  |
|  | *Adgre1* | -1.10445 | 0.044583 |  |
|  | *Cryba4* | -1.09187 | 7.27E-10 |  |
|  | *Ccl9* | -1.09139 | 0.005782 |  |
|  | *Ccl6* | -1.083 | 0.004053 |  |
|  | *Hc* | -1.07602 | 0.001946 |  |
|  | *Tex11* | -1.06382 | 0.091374 |  |
|  | *H2ac15* | -1.05257 | 0.029836 |  |
|  | *Il6ra* | -1.05239 | 0.028399 |  |
|  | *Chil3* | -1.03884 | 3.58E-06 |  |
|  | *Cbfa2t3* | -1.02208 | 1.15E-05 |  |
|  | *Csf2rb* | -1.01252 | 0.103902 |  |
|  | *Cntnap1* | -1.00722 | 0.134216 |  |
|  | *Insyn2b* | -1.00679 | 0.241194 |  |
|  |  |  |  |  |
|  |  |  |  |  |
|  |  |  |  |  |
|  |  |  |  |  |
|  |  |  |  |  |
| ***Up-regulated genes in Stat4-deficient ILC2*** | | | | |
|  |  |  |  |  |
|  | gene | log2FC | padj |  |
|  | *Capn11* | 7.959862 | 5.45E-13 |  |
|  | *Scn4b* | 3.733304 | 1.05E-10 |  |
|  | *Trpm1* | 2.83931 | 2.58E-05 |  |
|  | *Ston1* | 2.46955 | 1.51E-21 |  |
|  | *Popdc2* | 2.242675 | 9.12E-07 |  |
|  | *Ltbp1* | 2.235763 | 4.76E-07 |  |
|  | *Cd80* | 2.183062 | 1.5E-12 |  |
|  | *Tgfbr3* | 2.133472 | 7.71E-08 |  |
|  | *Adra2c* | 2.068207 | 2.33E-15 |  |
|  | *Gm15433* | 2.051021 | 2.52E-06 |  |
|  | *Tnfsf8* | 1.903247 | 6.55E-06 |  |
|  | *Wdfy1* | 1.869663 | 2.5E-211 |  |
|  | *Ifit3b* | 1.815078 | 1.5E-37 |  |
|  | *Gm7609* | 1.775013 | 2.46E-07 |  |
|  | *D630045J12Rik* | 1.748747 | 5.47E-06 |  |
|  | *Dipk1c* | 1.729478 | 0.0006 |  |
|  | *Dscaml1* | 1.670435 | 0.005822 |  |
|  | *Mx2* | 1.66332 | 6.27E-79 |  |
|  | *Prss35* | 1.637659 | 0.000454 |  |
|  | *Mx1* | 1.635017 | 1.25E-98 |  |
|  | *Tmem45a* | 1.620286 | 0.00048 |  |
|  | *Bcar3* | 1.576807 | 8.43E-15 |  |
|  | *Ifit3* | 1.565457 | 4.4E-112 |  |
|  | *Ifit1bl2* | 1.525464 | 1.1E-06 |  |
|  | *Ddx60* | 1.500291 | 3.6E-131 |  |
|  | *Oas1g* | 1.48995 | 2.96E-40 |  |
|  | *Ifit1bl1* | 1.470164 | 6.45E-72 |  |
|  | *Slfn1* | 1.434168 | 1.14E-64 |  |
|  | *Tph1* | 1.427816 | 6.46E-46 |  |
|  | *Ifi44* | 1.417912 | 6.3E-125 |  |
|  | *Glrp1* | 1.40127 | 0.002195 |  |
|  | *Rapgef3* | 1.396512 | 0.002871 |  |
|  | *Slfn5* | 1.384967 | 0 |  |
|  | *Oasl1* | 1.369001 | 2E-268 |  |
|  | *Ifit1* | 1.363277 | 2.7E-282 |  |
|  | *Apol8* | 1.355052 | 0.000119 |  |
|  | *Msc* | 1.328706 | 1.07E-09 |  |
|  | *Trim15* | 1.324467 | 0.006662 |  |
|  | *Palld* | 1.284582 | 0.036464 |  |
|  | *Gm12253* | 1.275481 | 2.69E-07 |  |
|  | *Phf11a* | 1.260068 | 5.74E-70 |  |
|  | *Usp18* | 1.258906 | 6.4E-163 |  |
|  | *Clip3* | 1.256792 | 0.001953 |  |
|  | *Gm16796* | 1.249204 | 0.003106 |  |
|  | *Xlr3a* | 1.240264 | 2.83E-05 |  |
|  | *Trim30c* | 1.221474 | 3.97E-07 |  |
|  | *Clgn* | 1.211527 | 0.000643 |  |
|  | *4930599N23Rik* | 1.207392 | 0.003978 |  |
|  | *Pkdcc* | 1.201551 | 3.19E-15 |  |
|  | *Apobec1* | 1.198706 | 2.69E-13 |  |
|  | *Gm2808* | 1.192956 | 4.16E-09 |  |
|  | *Penk* | 1.187992 | 0.009093 |  |
|  | *Gm20559* | 1.171369 | 1.3E-129 |  |
|  | *Tmcc2* | 1.165783 | 2.28E-06 |  |
|  | *Rtp4* | 1.159631 | 5.9E-204 |  |
|  | *Ldhd* | 1.154753 | 0.004197 |  |
|  | *Igsf9b* | 1.151003 | 0.015826 |  |
|  | *Oas2* | 1.148147 | 1.74E-53 |  |
|  | *Ust* | 1.138466 | 1.17E-05 |  |
|  | *Tlr7* | 1.125292 | 5.14E-08 |  |
|  | *Isg20* | 1.123796 | 1.31E-60 |  |
|  | *Irf7* | 1.11653 | 0 |  |
|  | *Oas1a* | 1.113893 | 4.5E-290 |  |
|  | *Isg15* | 1.108356 | 2.4E-123 |  |
|  | *Smoc1* | 1.106992 | 0.027446 |  |
|  | *Oasl2* | 1.094445 | 7.2E-106 |  |
|  | *Dhx58* | 1.089502 | 8.4E-192 |  |
|  | *Fat1* | 1.08628 | 0.0002 |  |
|  | *Il22* | 1.08387 | 0.03003 |  |
|  | *9930111J21Rik2* | 1.081664 | 1.9E-08 |  |
|  | *4930512H18Rik* | 1.080334 | 0.002958 |  |
|  | *Ifih1* | 1.07939 | 3.6E-128 |  |
|  | *Ifi213* | 1.075652 | 2.6E-106 |  |
|  | *Adgrg7* | 1.072441 | 6.63E-28 |  |
|  | *Slc41a2* | 1.070908 | 0.027357 |  |
|  | *Filip1* | 1.068554 | 0.000269 |  |
|  | *Eng* | 1.062546 | 1.93E-07 |  |
|  | *St6galnac2* | 1.056669 | 5.24E-11 |  |
|  | *Zfp985* | 1.050747 | 0.045427 |  |
|  | *Rsad2* | 1.049061 | 0 |  |
|  | *Sco1* | 1.0436 | 2.55E-88 |  |
|  | *Piwil4* | 1.039177 | 2.34E-07 |  |
|  | *Ly6a* | 1.033967 | 6.3E-278 |  |
|  | *Ttn* | 1.031445 | 8.31E-05 |  |
|  | *Ifi208* | 1.02589 | 5.7E-133 |  |
|  | *Ifi27l2a* | 1.024798 | 1.3E-256 |  |
|  | *Dll1* | 1.0167 | 5.25E-05 |  |
|  | *Nek6* | 1.013522 | 0.000295 |  |
|  | *Hif3a* | 1.008788 | 0.000474 |  |
|  | *Ifi214* | 1.004528 | 4.94E-79 |  |
|  | *Xaf1* | 1.003661 | 2E-145 |  |

**Table relative to Fig. 5F.** List of STAT4/STAT1 target genes identified in NK cells and their expression in ILC2 from IL-25-treated *Rag2^-/-^* and *Rag2^-/-^Stat4^-/-^* mice.

| ***STAT4/STAT1 regulated genes in NK cells from reference: PMID 30082830*** | | | | | | |
| --- | --- | --- | --- | --- | --- | --- |
|  |  |  |  |  |  |  |
|  |  | Log2 average expression in ILC2 | | | | |
|  |  |  |  |  |  |  |
|  |  |  | Gene | WT | KO |  |
|  |  |  | *Maf* | 13.43413 | 13.35459 |  |
|  |  |  | *Oas3* | 12.24061 | 13.11871 |  |
|  |  |  | *Rsad2* | 11.92369 | 12.97264 |  |
|  |  |  | *Slfn5* | 11.01923 | 12.40395 |  |
|  |  |  | *Oas1a* | 11.19326 | 12.30472 |  |
|  |  |  | *Cmpk2* | 11.5999 | 12.2598 |  |
|  |  |  | *Bst2* | 11.04254 | 11.92741 |  |
|  |  |  | *Ifit1* | 10.312 | 11.6721 |  |
|  |  |  | *Mx1* | 9.966383 | 11.60108 |  |
|  |  |  | *Mov10* | 11.11218 | 11.51738 |  |
|  |  |  | *Tspan32* | 11.44489 | 11.48861 |  |
|  |  |  | *Cited4* | 10.78355 | 10.87046 |  |
|  |  |  | *Ifih1* | 9.788816 | 10.86412 |  |
|  |  |  | *Parp12* | 9.815512 | 10.74687 |  |
|  |  |  | *Oasl2* | 9.501756 | 10.59628 |  |
|  |  |  | *Tgtp2* | 9.886375 | 10.28428 |  |
|  |  |  | *Ddx60* | 8.662076 | 10.17777 |  |
|  |  |  | *Tor3a* | 9.331134 | 10.04749 |  |
|  |  |  | *Grn* | 9.737088 | 9.919967 |  |
|  |  |  | *Iigp1* | 9.863116 | 9.864032 |  |
|  |  |  | *Ifit3* | 8.19687 | 9.754602 |  |
|  |  |  | *Ccrl2* | 9.509166 | 9.736302 |  |
|  |  |  | *Mx2* | 7.932249 | 9.598165 |  |
|  |  |  | *Cd74* | 9.055263 | 9.581427 |  |
|  |  |  | *Isg20* | 8.387927 | 9.499158 |  |
|  |  |  | *Sdc3* | 8.515968 | 9.467986 |  |
|  |  |  | *Gpsm2* | 9.467577 | 9.424882 |  |
|  |  |  | *Oas1c* | 8.745762 | 9.462835 |  |
|  |  |  | *Oas2* | 8.220659 | 9.367149 |  |
|  |  |  | *Ifit2* | 8.341551 | 9.318395 |  |
|  |  |  | *Slfn1* | 7.747041 | 9.19748 |  |
|  |  |  | *Themis2* | 9.112832 | 9.075771 |  |
|  |  |  | *Ifit1bl1* | 7.601699 | 9.071968 |  |
|  |  |  | *Parp11* | 8.445376 | 8.554196 |  |
|  |  |  | *Tgtp1* | 8.406586 | 8.405655 |  |
|  |  |  | *Oas1g* | 6.820545 | 8.303672 |  |
|  |  |  | *Frmd4a* | 7.657855 | 8.074424 |  |
|  |  |  | *Hmgn3* | 7.428771 | 8.058808 |  |
|  |  |  | *Atf3* | 7.451952 | 7.937892 |  |
|  |  |  | *Ifit3b* | 6.026217 | 7.843813 |  |
|  |  |  | *Capn5* | 7.725463 | 7.709163 |  |
|  |  |  | *Gm12185* | 7.142732 | 7.385478 |  |
|  |  |  | *Gm8369* | 6.229411 | 7.169063 |  |
|  |  |  | *Ms4a4c* | 6.484708 | 6.869842 |  |
|  |  |  | *BC051226* | 6.116367 | 6.745415 |  |
|  |  |  | *Axl* | 6.303874 | 6.303205 |  |
|  |  |  | *Gm4841* | 6.046039 | 6.246183 |  |
|  |  |  | *Csf1* | 5.955885 | 6.069003 |  |
|  |  |  | *Slc6a9* | 5.746812 | 5.064678 |  |
|  |  |  | *Gm5431* | 4.548065 | 5.475856 |  |
|  |  |  | *Ifit1bl2* | 3.864723 | 5.449409 |  |
|  |  |  | *Ajuba* | 5.32229 | 4.910567 |  |
|  |  |  | *Cdkl4* | 4.315758 | 4.970427 |  |
|  |  |  | *Fcgr2b* | 4.756485 | 3.025518 |  |
|  |  |  | *Btbd16* | 4.355491 | 4.756179 |  |
|  |  |  | *Serpina3f* | 3.812339 | 4.395653 |  |
|  |  |  | *F830016B08Rik* | 3.70325 | 4.291372 |  |
|  |  |  | *Trib3* | 4.278409 | 4.21032 |  |
|  |  |  | *S100a9* | 3.950736 | 3.541498 |  |
|  |  |  | *Dmtn* | 3.85357 | 3.192648 |  |

**Table relative to oligonucleotides used for qRT-PCR**

| Oligonucleotides | Sequence | Assay |
| --- | --- | --- |
| Mouse IFN-beta FOR | GCCTTTGCCATCCAAGAGATGC | qRT-PCR |
| Mouse IFN-beta REV | ACACTGTCTGCTGGTGGAGTTC | qRT-PCR |
| Mouse IFN-alpha FOR | CACTGTGTACCTGAGAGAGAAGAAAC | qRT-PCR |
| Mouse IFN-alpha REV | GGAAGACAGGGGCTCTCCAGACT | qRT-PCR |
| GAPDH FOR | TCG TCC CGTAGACAAAATGG | qRT-PCR |
| GAPDH REV | TTGAGGTCAATGAAGGGGTC | qRT-PCR |
